# Efficient Physical Embedding of Topologically Complex Information Processing Networks in Brains and Computer Circuits

**DOI:** 10.1371/journal.pcbi.1000748

**Published:** 2010-04-22

**Authors:** Danielle S. Bassett, Daniel L. Greenfield, Andreas Meyer-Lindenberg, Daniel R. Weinberger, Simon W. Moore, Edward T. Bullmore

**Affiliations:** 1 Department of Physics, University of California Santa Barbara, Santa Barbara, California, United States of America; 2 Institute for Collaborative Biotechnologies, University of California Santa Barbara, Santa Barbara, California, United States of America; 3 Behavioral & Clinical Neurosciences Institute, Department of Psychiatry, University of Cambridge, Cambridge United Kingdom; 4 Computer Laboratory, University of Cambridge, Cambridge, United Kingdom; 5 Central Institute of Mental Health, Mannheim, Germany; 6 Genes, Cognition, and Psychosis Program, Clinical Brain Disorders Branch, National Institute of Mental Health, Bethesda, Maryland, United States of America; University College London, United Kingdom

## Abstract

Nervous systems are information processing networks that evolved by natural selection, whereas very large scale integrated (VLSI) computer circuits have evolved by commercially driven technology development. Here we follow historic intuition that all physical information processing systems will share key organizational properties, such as modularity, that generally confer adaptivity of function. It has long been observed that modular VLSI circuits demonstrate an isometric scaling relationship between the number of processing elements and the number of connections, known as Rent's rule, which is related to the dimensionality of the circuit's interconnect topology and its logical capacity. We show that human brain structural networks, and the nervous system of the nematode *C. elegans*, also obey Rent's rule, and exhibit some degree of hierarchical modularity. We further show that the estimated Rent exponent of human brain networks, derived from MRI data, can explain the allometric scaling relations between gray and white matter volumes across a wide range of mammalian species, again suggesting that these principles of nervous system design are highly conserved. For each of these fractal modular networks, the dimensionality of the interconnect topology was greater than the 2 or 3 Euclidean dimensions of the space in which it was embedded. This relatively high complexity entailed extra cost in physical wiring: although all networks were economically or cost-efficiently wired they did not strictly minimize wiring costs. Artificial and biological information processing systems both may evolve to optimize a trade-off between physical cost and topological complexity, resulting in the emergence of homologous principles of economical, fractal and modular design across many different kinds of nervous and computational networks.

## Author Summary

Brains are often compared to computers but, apart from the trivial fact that both process information using a complex physical pattern of connections, it has been unclear whether this is more than just a metaphor. In our work, we rigorously uncover novel quantitative organizational principles that underlie the network organization of the human brain, high performance computer circuits, and the nervous system of the nematode *C. elegans*. We show through a topological and physical analysis of connectivity data that each of these systems is cost-efficiently embedded in physical space; they are organized as economical modular networks, paying a modest premium in wiring cost for the functional advantages of high dimensional topology. We also show that the fractal properties of human brain network connectivity can be used to explain allometric scaling relations between grey and white matter volumes in the brains of a wide range of differently sized mammals—from mouse opossum to sea lion—further suggesting that these principles of nervous system design are highly conserved across species. We propose that market-driven human invention and natural selection have negotiated trade-offs between cost and complexity in design of information processing networks and convergently come to similar conclusions.

## Introduction

Since the publication of Watts and Strogatz's seminal article, “Collective dynamics of ‘small-world’ networks”, network science, as it has now come to be called, has extensively pervaded the scientific community, transcending previously impermeable boundaries between disciplines at every turn [1]. The availability of integrative tools to quantify the emergent behavior of systems made up of many interacting parts, whether they be people in a social network, proteins in a protein-interaction network, or individual web pages in the WWW, allowed many disciplines to add an entirely new level of description and find common ground with traditionally unrelated fields. The beauty of the topological formalism stemmed from its simplicity: each connection between two parts of the system was indicated by a line of unitary length, collectively giving an understanding of connectivity structure in the abstract, e. g. in topological space. While this strong focus on interconnect topology has enabled seminal discoveries in a wide variety of networks in the past decade, it inevitably neglects a fundamental property of the majority of these systems: their existence in a physical space. Gene co-expression profiles have specific anatomical distributions throughout the body; proteins have spatial distributions within cells that may increase or decrease the probability of their interactions; humans have physical locations that may influence who they make friends with; countries have frontiers with each other that may affect their trade of goods. Each of these complex systems can be described by a network topology that is highly dependent on each node's physical location. Indeed, understanding the importance of physical node placement in network growth and resultant topologies is an active topic of research in network science [2], [3].

At this timely juncture, we investigate the interdependence of topology and physicality in a “topophysical” analysis of information processing networks. High performance computer circuits have been empirically observed to exhibit a simple scaling relationship, known as Rent's rule, between the number of nodes or “gates” in any piece of the circuit and the number of connections (inputs or outputs) to that piece of circuit or “block of logic”, over a range of spatial scales [4]. First observed by Rent in the 1960s, this scaling relationship has held up remarkably well as circuits have evolved rapidly in terms of size and functional performance. Circuits with greater logical capacity have higher values of the Rent exponent, indicating more complex wiring or higher dimensionality of the interconnect topology of the circuit. Rentian scaling is one aspect of fractal or self-similar network design principles that are also reflected in the hierarchical modularity of VLSI circuits, which typically consist of “modules-within-modules”. Minimization of the cost of wiring VLSI circuits has been an important economic factor in their commercial evolution. Rentian scaling represents a cost-efficient solution to the challenge of embedding a high dimensional functional interconnect topology in a relatively low dimensional physical space with economical wiring costs [5], [6].

Given the mounting evidence that many complex systems share important organizational properties in common [7], we hypothesized that other informational systems, which have evolved by natural selection rather than technological development, would also be characterized by high dimensional fractal topologies mapped cost-efficiently into physical space. The hypothesis that biological and artificial information processing systems, in particular, might share network properties such as hierarchical modularity that confer adaptivity or evolvability of function [8] dates back to Simon's prescient analysis [9] but has not yet been extensively tested using contemporary datasets and network analysis tools. Here we study the only complete neuronal connectome currently available, that of the nematode worm *Caenorhabditis elegans*, as well as large-scale human brain structural networks recently derived from neuroimaging data (using both magnetic resonance imaging, MRI, and diffusion spectrum imaging, DSI), and a benchmark VLSI circuit.

## Results

We investigated the topological and physical properties of three distinct and differently sized information processing networks - two biological nervous systems and an artificial computer system. The two biological systems were the human brain structural network and the neuronal connectome of the nematode worm *Caenorhabditis elegans*. Human brain structural networks were measured at a relatively coarse-grained, regional level of resolution (cm) using two complementary neuroimaging techniques in two different samples; see [10] for a review of graphical methods of network analysis in human neuroimaging. Covariation of regional gray matter volumes was measured using conventional MRI data on a sample of 259 healthy volunteers and a binary network was constructed by thresholding the inter-regional partial correlation matrix [11]. Gray matter covariation has previously been proposed as an indirect marker of anatomical (axonal) connectivity between regions [12], [13] and the rationale is rehearsed in supplementary Text S1. As an alternative and more direct measure of anatomical connectivity, we also estimated the connection probabilities between regions by tractographic analysis of diffusion spectrum imaging (DSI) data on 5 healthy volunteers [14]. This approach allows us to construct an anatomical network, by thresholding the inter-regional connection probability matrix, for each individual participant; whereas the approach based on inter-individual covariation of gray matter volumes in conventional MRI yields only a single network for the whole group of participants. The nervous system of *C. elegans* has been precisely measured at a finer-grained, cellular (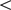
mm) level of resolution [15], [16] and is highly reproducible across individual worms. The computational system was a benchmark very large scale integrated (VLSI) circuit (ISCAS89 sequential logic circuit s953 [17]).

**Figure 1:**
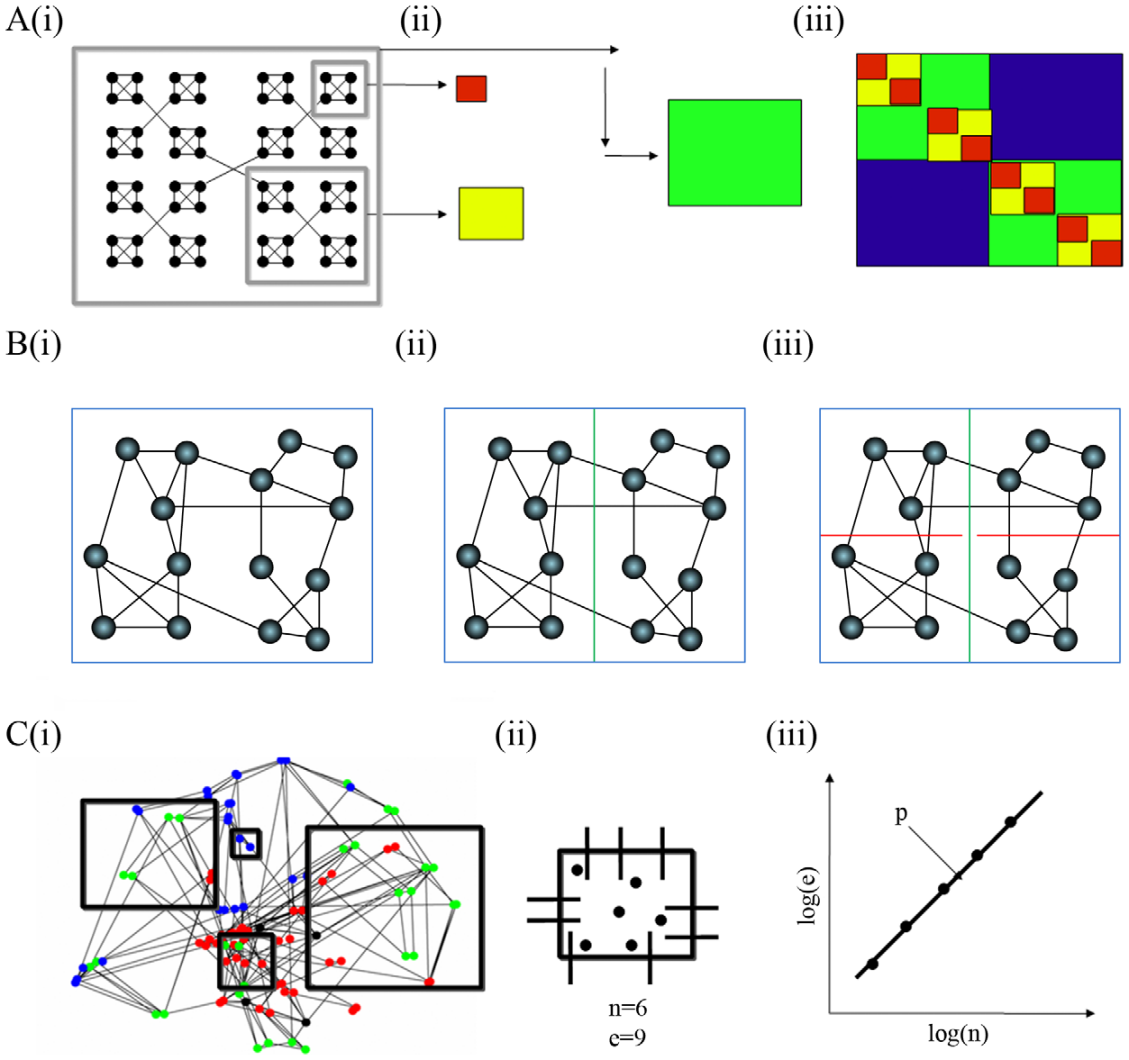
Schematic of some key methods. A hierarchical modular network, A(i), is made up of modules, A(ii) green, which are made up of sub-modules, A(ii) yellow, and sub-sub-modules, A(ii) red, which are collectively visualized by a co-classification matrix, A(iii), where hierarchical modularity is evident by layers of color located along the diagonal [74]. To estimate the topological Rent exponent and dimension of a network, B(i), we first cover the network with a single box large enough to cover it entirely; then we recursively partition the box (B(ii) and B(iii)) into halves, quarters, and so on using a partition algorithm that minimizes the number of edges cut by each partition. For each iteration of this process, we count the number of nodes within a partition (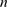), and the number of edges (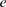) crossing the partitions; a linear relationship between these two variables plotted on logarithmic axes indicates topological Rentian scaling of network connectivity and provides an estimator of the topological dimension 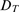 of the network[21], [22]. To estimate the physical Rent exponent, we randomly place 5000 randomly sized boxes on the physically embedded network, e.g., the human brain network in anatomical space C(i). Then we count the number of nodes 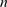 and the number of boundary-crossing edges 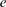 for each box C(ii) and estimate the physical Rent exponent 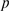 by the linear relationship between these two variables on logarithmic axes, C(iii).

We applied methods of network analysis, drawn mainly from the literature on VLSI design, consistently to both nervous and computational systems; see Materials and Methods and Table 1 for details, and Figure 1 for a graphical depiction of analysis methods.

**Table 1:** Measures of fractal connectivity and physical embedding in computational and nervous systems.

Network	Nodes	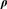	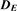	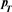	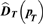	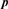	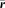	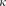
**Observed**								
VLSI	440	0.4%	2	0.73 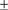 0.04	3.81 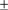 0.64	0.901 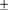 0.006	7.78	1.68
*C. elegans*	277	2.7%	3	0.77 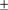 0.06	4.42 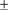 1.53	0.74 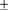 0.07	53.34	28.39
Human brain (MRI)	104	15%	3	0.75 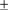 0.07	4.12 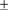 1.55	0.828 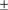 0.005	3.02	1.60
Human brain (DSI)	1000	2.7%	3	0.78 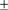 0.07	4.54 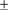 2.12	0.782 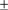 0.014	4.55 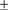 0.33	1.97 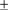 0.20
**Randomly rewired**								
VLSI	440	0.4%	2	0.81 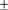 0.06	5.26 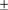 2.42	0.927 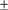 0.003	10.90	1.98
*C. elegans*	277	2.7%	3	0.79 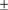 0.05	4.76 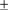 1.48	0.805 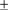 0.003	88.43	44.54
Human brain (MRI)	104	15%	3	0.82 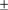 0.06	5.55 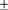 1.06	0.874 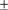 0.003	12.00	6.27
Human brain (DSI)	1000	2.7%	3	0.86 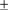 0.05	7.14 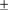 2.77	0.925 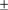 0.002	10.88	3.23
**Minimally rewired**								
VLSI	440	0.4%	2	0.46 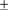 0.06	1.85 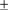 0.23	0.509 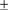 0.005	1.21	0.43
*C. elegans*	277	2.7%	3	0.43 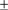 0.28	1.75 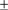 1.69	N/A	1.34	1.36
Human brain (MRI)	104	15%	3	0.59 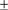 0.13	2.43 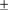 1.13	0.93 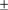 0.01	1.88	1.47
Human brain (DSI)	1000	2.7%	3	0.57 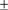 0.11	2.32 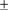 0.79	0.68 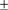 0.004	2.17	1.01

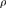
 connection density; 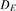, Euclidean (embedding) dimension; 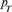, observed topological Rent's exponent; 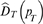, fractal dimension of network topology estimated from the topological Rent's exponent, 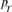; 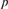, observed physical Rent exponent; 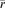, mean connection distance between nodes (Eq 6); 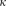, measure of cost-efficient embedding (Eq 4). Errors reported are 95% confidence intervals for fits of 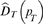, 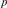; and standard deviation over individual subject's values for the DSI estimations of 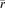 and 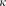. For the DSI estimations of 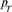, 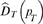, and 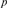, combined errors (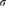) are reported which include the errors of fit (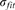) and individual variation (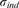) as given by 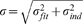 (see supplementary Text S1 for results from individual DSI scans). Values for the randomly rewired networks are averages over 10 random network instantiations.

### Hierarchical modularity

**Figure 2:**
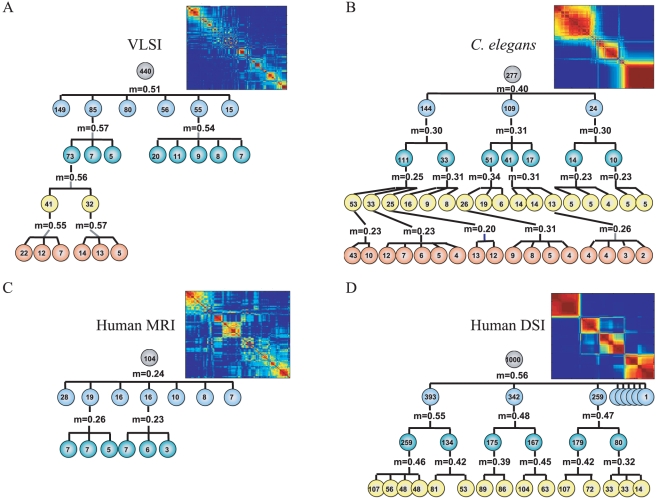
Hierarchical modularity in nervous and computational systems. Dendrograms displaying significant modular and sub-modular structure for (*A*) a very large scale integrated circuit, (*B*) the nematode worm *C. elegans*, (*C*) the human cortical anatomical network estimated using conventional MRI in 259 normal volunteers and (*D*) the human cortical anatomical network estimated using diffusion spectrum imaging (DSI) from an independent sample of 5 normal volunteers. The modularity, 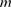, of each of these matrices was estimated using the Louvain community detection algorithm [18]; 1-tailed t-tests were performed to determine where the modularity of the observed network was higher than the modularity of a functional random (p-value, 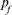), and pure random (p-value, 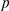) network. The matrices were decomposed into their sub-modules, and each sub-module was tested for modularity, 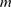, greater than functional and pure random networks (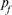, 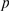) of the same size as the module being tested. This process was iteratively performed: sub-modules were tested for non-random modularity, and if sub-sub-modules were identified in this way then each of them was in turn tested for non-random modularity. All modules shown in the decomposition had 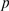, 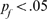 except for those few indicated in gray (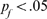) and blue (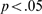). Complete decompositions are shown for the VLSI and human brain MRI network; both the *C. elegans* and human brain DSI networks continue to deeper hierarchical levels, here not shown due to space constraints (see supplementary Text S1 for full decompositions). *Insets* The inset panels give a visual depiction of the hierarchical modularity of each system, which has been represented by a co-classification matrix where red/brown colors highlight modules or clusters of nodes with high local interconnectivity and relatively sparse connectivity to nodes in other modules [74]; see also Figure 1 for a schematic.

All three information processing networks demonstrated modularity of community structure, such that each network could be sub-divided into a number of sparsely interconnected modules each comprising a number of densely intra-connected nodes. Indeed, we found that there were often “modules within modules”, such that community structure was present on a hierarchy of topological scales. This property of hierarchical modularity can be discerned simply by inspection of the co-classification matrix of each network (Figure 2 *left*) but is more robustly demonstrated by the results of iterative modular decomposition using the Louvain algorithm [18], [19] (Figure 2 *right*). The *C. elegans* nervous system and the VLSI circuit both had significantly non-random modularity over 4 hierarchical levels. The human brain network derived from DSI data was significantly modular over 3 hierarchical levels and the network derived from conventional MRI data was modular over 2 levels; see supplementary Text S1 for additional results. It is important to note that such hierarchical modularity is consistent with a fractal or scale-invariant topology of connections between elements of the systems [5], [6].

### Rentian scaling

In the development of VLSI circuits, a simple power law, known as Rent's rule, has been discovered to define the scaling relationship between the number of external signal connections 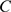 to a block of logic and the number of connected nodes 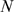 in the block [4]:
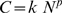
(1)where 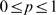 is the Rent exponent and 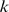 is the Rent coefficient.

Moreover, this scaling relationship can be measured in both physical space and topological space by defining a ‘block’ as either a physical box or a topological partition; see Figure 1 and Materials and Methods for details. We will refer to the Rent exponent estimated in physical space as the physical Rent exponent, denoted simply 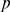; and we will refer to the Rent exponent estimated in topological space as the topological Rent exponent, denoted 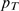. As we show below, the topological Rent exponent can be used to estimate the fractal dimension of the network topology, and can be compared to the physical Rent exponent to assess the cost-efficiency with which the network has been embedded in a Euclidean dimensional space.

#### Topological Rentian scaling and fractal dimension

Topological Rentian scaling is generally defined as the scaling of the number of nodes 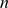 within a topological partition with the number of connections or edges, 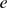, crossing the boundary of that topological partition. If these two variables scale with each other in log-log space, the network is said to show topological Rentian scaling or fractal topology. The exponent of this scaling relationship is known as the topological Rent exponent, 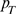 and is related to the topological dimension, 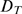, of the network according to 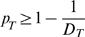 [5]. Thus higher values of the topological Rent exponent are indicative of higher dimensional network topology.

**Figure 3:**
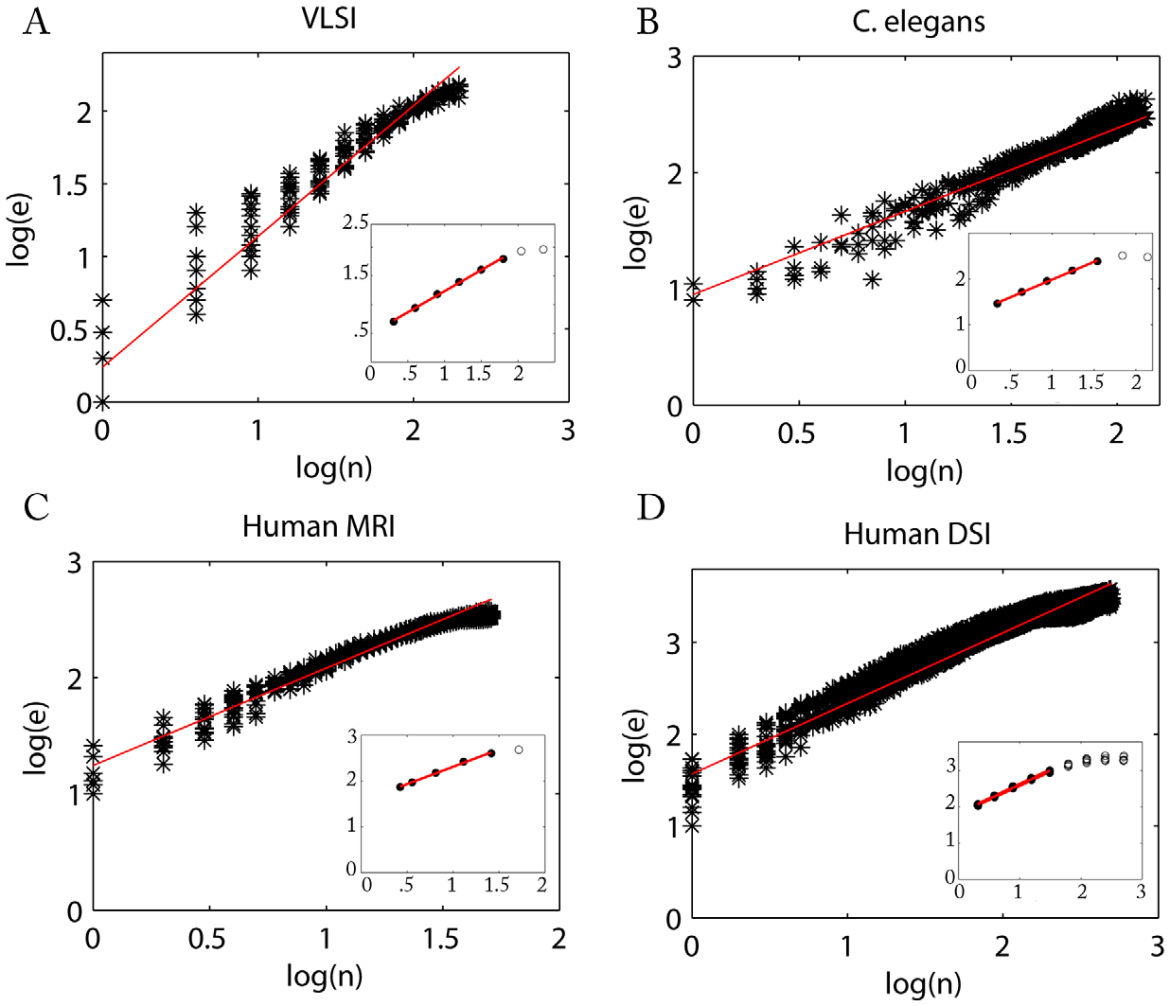
Topological and physical Rentian scaling in nervous and computational systems. Physical Rentian scaling in (*A*) a very large scale integrated circuit, (*B*) the nematode worm *C. elegans*, (*C*) the human cortical anatomical network estimated using conventional MRI in 259 normal volunteers and (*D*) the human cortical anatomical network estimated using diffusion spectrum imaging (DSI) from an independent sample of 5 normal volunteers, is shown by a power law scaling of the number of connections or edges (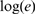) and number of processing elements (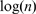) in a physical box; data points for each physical box are shown by black stars. The Rent exponents for each system were estimated by the gradients of the fitted red lines (see Table 1). Note: Data and linear shown in *D* are for a single subject. *Insets* Topological Rentian scaling in nervous and computational systems is shown by a power law scaling of the number of nodes (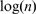) in a topological partition and the number of edges crossing the boundary of that partition (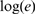); data points for each topological partition are shown by black circles. The network topology of each system was iteratively partitioned in topological space. All networks contained a linear scaling regime (so-called Region I, filled black circles) and a regime at larger partition sizes where linear scaling broke down due to boundary effects (so-called Region II, empty black circles). The slope, 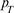, of the line through points within Region I was estimated using a weighted linear regression (red line); see Table 1. Note: Data and linear fits for all six DSI scans are shown in *D*.

**Figure 4:**
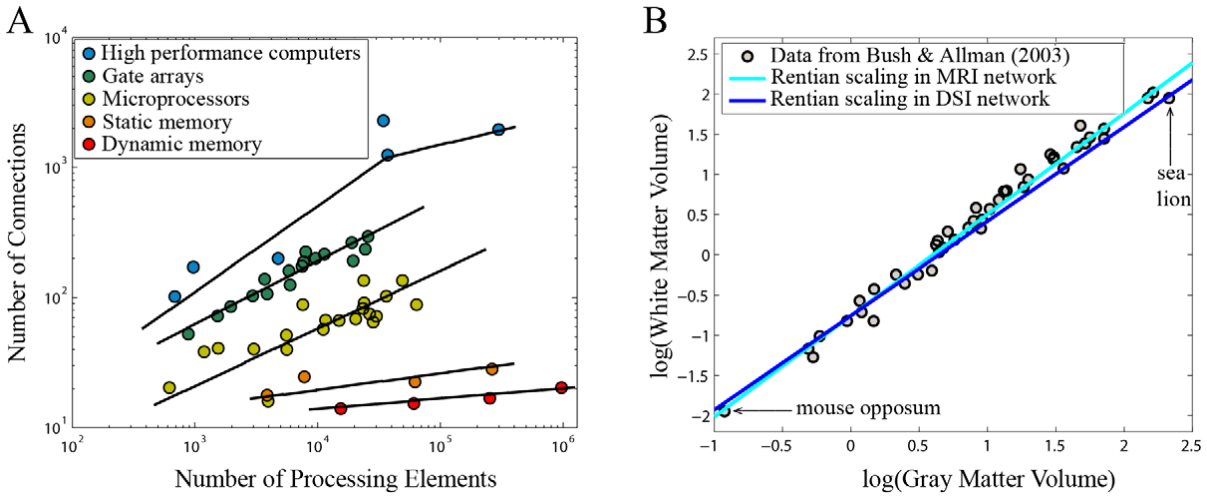
Allometric and Rentian scaling of VLSI circuits and mammalian brains. In computer circuits (A), the number of connections at the boundary of a chip scales in a Rentian power law with the number of processing elements; the Rent exponent 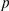 is greater for high performance computers (shown in blue) than for simpler dynamic memory circuits (shown in red); see pp. 416–421 in [76] for data values plotted here. In the cerebral hemispheres of mammalian brains (B), there is an allometric scaling relationship between white matter volume (related to connectivity of elements) and gray matter volume (related to number of processing elements); see Table 1 in [26] for data values plotted here. The exponent of this volume scaling relationship over species, 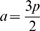, is simply related to the Rent exponent of mammalian cerebral connectivity, 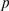. Lines fitted through the intercept of the data show the allometric scaling relationship predicted by the Rent exponent estimated for neuroimaging data on a single species (*Homo sapiens*), using MRI (cyan, 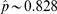) and DSI (blue, 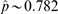) (Table 1). Errors in the fits are smaller than the line width.

We found that all information processing networks demonstrated topological Rentian scaling (see Table 1 and Figure 3). For all networks, a power law provided a better fit to the topological data on 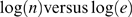 than comparable exponential, linear or polynomial models; see supplementary Text S1. It was also notable that all the networks had a topological dimension 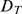 greater than the Euclidean dimension 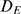 in which they were physically embedded. This inequality indicates that the dimensionality of connections between nodes of information processing networks is generally greater than one would expect for a 2- or 3-dimensional lattice (for which 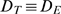). In the evolution of VLSI circuitry, progressively higher dimensional interconnections between logic gates have been associated with greater logical capacity; see Figure 4. However, the development of high dimensional network topologies necessarily comes at a cost in terms of wiring. The wiring cost of a network with 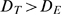 will inevitably be greater than the absolute minimum cost of wiring an equivalent sized network with 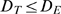 (e.g., a regular lattice). Indeed, it is nontrivial (NP-complete) to find the physical layout of a high dimensional topology which optimally minimizes its wiring cost [20].

These findings were supported by the results of estimating fractal dimension of the network topology by an alternative, box-counting estimator [21], [22], which provided consistent estimates of 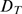, as discussed in the supplementary Text S1.

#### Physical Rentian scaling and efficient embedding

The physical Rent exponent is estimated from the scaling of the number of nodes 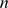 within a physically located subset with the number of connections or edges, 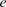, crossing the boundary around the nodes; see Figure 1 and Materials and Methods. For a given network topology, the minimum possible physical Rent exponent, 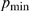, associated with the most efficient possible physical placement, is theoretically related to the topological Rent exponent, 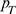, as follows [23]:
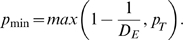
(2)For all information processing networks, a power law provided a better fit to the physical data on 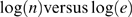 than comparable exponential, linear or polynomial models; see supplementary Text S1. In all cases, we found that 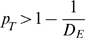 (which is 2/3 or 0.67 for 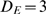 and 0.5 for 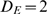). Therefore, the minimum physical Rent exponent possible for each of these networks was equal to its topological Rent exponent: 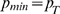. As shown in Table 1, the observed physical Rent exponent, 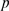, was indeed close to its theoretically predicted minimum value, 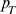, indicating that both biological and computational networks had been cost-efficiently embedded in physical space.

#### Wiring length

From the theory of VLSI design, we also know that the mean connection distance 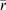 between nodes in a circuit is related to its size 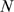, topological dimension 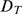 and embedding dimension 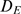 by the equation [5], [24]

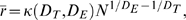
(3)where the coefficient 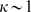 if the network has been cost-efficiently embedded. As shown in Table 1, all information processing networks, especially the human brain networks, had values of 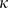 close to unity, providing additional evidence in favor of their cost-efficient embedding. The relatively large value of 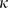 for the *C. elegans* system reflects the fact that its connections are physically extended to innervate an entire organism.

**Figure 5:**
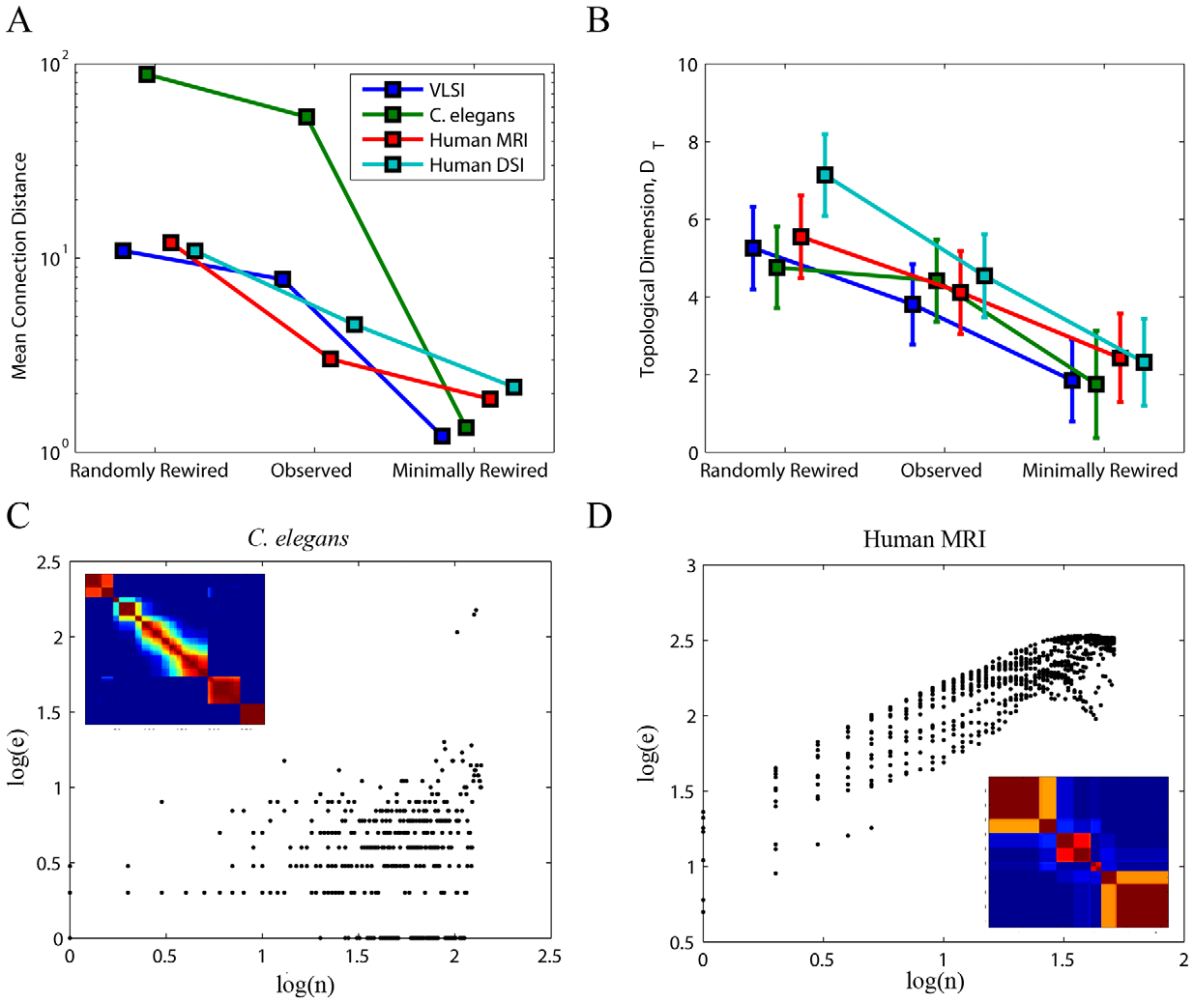
Minimally rewired networks. Wiring costs of nervous and computational networks are lower than expected in a random network, but can be reduced by a rewiring algorithm designed to minimize connection distance between elements (A); see Table 1. However, the fractal dimension of network topology, while lower than expected in a randomly rewired network, is also reduced by rewiring for cost minimization (B). Rentian scaling is either destroyed or disrupted by minimally rewiring the *C. elegans* connectome (C) or the human MRI network (D). The inset panels in (C,D) show the hierarchical modularity of each minimally wired system, which has been represented by a co-classification matrix as in Figure 2.

Although these information processing networks were efficiently embedded, they were not wired for absolutely minimum cost. We rewired each circuit to minimize its wiring cost and compared the cost and topological dimension of this minimally wired version of the network to its observed cost and dimensionality. In all cases, we found that wiring cost could be further reduced by a minimization algorithm, indicating that cost had not been minimized by selection pressures on the observed networks. However, such wiring minimization could only be achieved at the expense of a reduced topological dimension (see Table 1 and Figure 5).

### Allometric scaling

In VLSIs, the self-similar nature of Rent's rule is used to predict the effect of scaling the system, by keeping the exponent and coefficient the same. Indeed, despite the lack of any explicit imperative for individual circuit designers to follow this law, Rent exponents can be a remarkably reliable predictor for emergent VLSI allometric scaling properties, over many orders of magnitude, such as can be seen in Figure 4A.

We considered the related question of whether the Rentian scaling of connections between cortical regions in the human brain networks could be related to the allometric scaling of gray matter 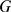 and white matter 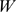 volumes previously described over a wide range of differently sized mammalian species, from mouse opposum to sea lion [25], [26] (Figure 4). It can be shown that the strong power law relationship 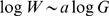 has an allometric exponent 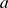 that is simply related to the physical Rent exponent 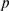 of mammalian cortical networks, i.e., 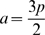; see Materials and Methods and supplementary Text S1 for details. On this basis, we used the estimate of 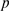 for human anatomical networks measured using MRI, 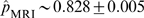, and using DSI, 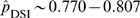, to predict the allometric scaling relationship between cerebral gray and white matter (Figure 4). The 95% confidence interval on the slope of the true data leads to a range of 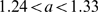 while the interval estimated from the MRI network was 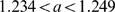 and for the DSI networks 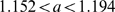 (the slight underestimation of 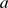 from DSI data may be related to a measurement bias against long distance connections in DSI-based tractography; see supplementary Text S1). The quality of prediction of mammalian allometric scaling from Rent exponents estimated in a single species is consistent with the idea that mammalian cortical networks are generally connected in accordance with the same Rent exponent, and this constrains the allometric scaling relations between gray and white matter volume which emerge over differently sized species.

To make the same point a different way, we converted previously reported allometric scaling exponents for the cerebral cortex and cerebellum [26] to the corresponding Rent exponents and fractal dimensions. Prior estimates of the cortical allometric scaling exponent 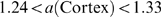 correspond to an interval of Rent exponents 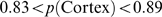 which includes the empirical estimates from human MRI data (Table 1); whereas the prior cerebellar allometric scaling exponents 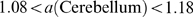 correspond to an interval of Rent exponents 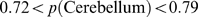. Although we were unable to verify the allometrically predicted Rent exponents for human cerebellum (because the MRI datasets did not include cerebellar measurements), we note that the prediction of larger Rent exponents and fractal dimensions for human cortical compared to cerebellar systems is consistent with the arguably greater logical capacity of cortical systems and the observation from VLSI circuits that higher dimensional connectivity is associated with greater logical capacity; see Figure 4. The hypothesis that cerebellar Rent exponents are indeed significantly reduced compared to cortical systems, and the more general idea that Rent exponents of neural systems are functionally related to their logical capacity, demand further direct investigation in future.

## Discussion

We have shown that several topological and physical principles of network organization are quite consistently represented across a wide range of different information processing systems. Artificially generated computer circuits and biologically evolved nervous systems are generally fractal modular networks that represent the cost-efficient physical embedding of a high dimensional interconnect or connectome topology in a lower dimensional Euclidean space. These properties help to explain the allometric scaling relationship between grey and white matter volumes across differently sized mammalian species and they provide fresh insight into why the wiring costs of nervous systems are not more strictly minimized.

In the remainder of this discussion, we will attempt to locate these key findings in the context of both past and present biological descriptions, computational models, and analysis methods.

### Hierarchical modularity

Modularity is a fundamental and protean word with many meanings in neuroscience. Psychological or functional modularity refers to separability or informational encapsulation of cognitive processes, which may be neurally represented by specialist, localised processing centres in the brain. It is one of the key ideas behind phrenology and faculty psychology [27]. There is also a well articulated neurodevelopmental aspect of modularity. For example, the embryonic development of chick hindbrain follows a strict chronological progression of cellular differentiation from caudal to rostral modules of nervous tissue, called rhombomeres, each of which comprises cells that share distinctive patterns of genetic co-expression compared to cells in neighbouring tissue modules [28]. Here we are concerned with topological modularity [18], [29] - a more general and quantitative version of the concept - that we have applied to analysis of information processing network organization.

Topological modularity is sometimes also referred to as the community structure of a network because it decomposes the global network into a set of modules or communities each comprising nodes that are densely intra-connected with each other and relatively sparsely inter-connected to nodes in other modules. This basic design of sub-systems within the global system is functionally advantageous in various ways. As Herbert Simon argued originally, the key advantage of such a design for an information processing network is that it confers rapid adaptivity or evolvability: the system can evolve or adapt to new information one module at a time, without risking loss of function in modules that are already well-adapted. For this reason, Simon predicted that all “physical symbol processing systems” would share a general architecture of complexity, including modularity (or near-decomposability as he sometimes called it) as a key principle [9]. Our results are compatible with Simon's prediction - all the information processing systems we considered could be decomposed into modules, or indeed “modules within modules”. For a classically modular decomposition, the system is decomposed into a single lower level of organization in terms of multiple sub-systems. For a hierarchically modular decomposition, the system is iteratively decomposed into multiple nested lower levels of organization in terms of sub-systems, sub-sub-systems, etc. When modularity is expressed consistently at several scales, we can describe the system as hierarchically modular. This property was clearly seen for the VLSI circuit, *C. elegans* and human connectomes, for each of which modularity was expressed consistently across several (4 or more) topological scales, so the system as a whole could be described in terms of sub-sub-sub-systems, or even lower hierarchical levels in the case of *C. elegans* and the human DSI network (see supplementary Text S1). Even the smallest and least precisely estimated connectome, that derived from the human MRI datasets, also generated networks with some hierarchical modular properties (across 2 topological scales) and this observation is compatible with other data demonstrating hierarchical modularity in human brain functional networks derived from “resting state” functional MRI data [30].

The observation that nervous systems generally share a hierarchically modular topology is particularly relevant to the question of how they support function. As is perhaps intuitive, there is mounting evidence that the modular architecture of anatomical structure determines the possible emergent functions of the network under study [31], [32]. Functional patterns on hierarchically modular architectures have specifically been shown to display computationally advantageous dynamics characterized by stability and diversity [33], unlike simulated dynamics on either random or non-hierarchically small-world architectures [34]. Sporns provides a simple generative model for fractal hierarchical networks and shows further relationships between their structural and functional properties, suggesting that connectivity may strongly constrain dynamics [32], [35]. Computational models of hierarchical modularity have shown that networks configured in this way have the distinctive advantage of being robustly stable under large scale reconnection of substructure [36].

Overall, we find there is strong empirical evidence, convergent with prior theoretical and computational results, for fractal modularity of information processing networks. We now consider some other aspects of the scale invariance of these systems.

### Fractal dimensions and wiring costs

Fractal dimensions are most frequently encountered in analysis of the physical properties of some rough, irregular process in space or time. A famous example is the fractal dimension of the fjord-riven western coastline of Norway (1.52) which is considerably greater than the Euclidean dimension of a straight line (1) but less than the 2 dimensions of the Euclidean plane in which the coastline is embedded on the page of an atlas. This is a fractional or non-integer measure of the dimensionality of a geometric system, 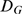, and it will generally be less than or equal to the integer dimensional Euclidean space in which the process is embedded. Thus the irregular convolutions of sulco-gyral folding in the human brain are associated with fractal dimension of the cortical surface 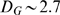, greater than a smooth 2D plane but less than the 3-dimensional volume in which the brain is embedded. Although its estimation is a matter of ongoing methodological research [37], [38], the fractal dimension of the cortical surface has already been used to describe healthy and abnormal neurodevelopment [39], [40] as well as aging [41].

However, here we have been concerned with a related but different measure: the fractional dimension of a topologically defined system, 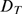. The dimension of a topology is a non-integer measure of the complexity of the interconnections between nodes, regardless of their physical location, and is therefore not constrained by the dimensionality of the physical embedding space. The dimension of a topology can range from 0 to infinity, in the limiting case of a very large, perfectly random network. If the network is embedded in a physical space, its topological dimension may therefore be larger than its embedding dimension, which in real space is at most 3. We found convergent evidence, by two independent estimators, for information processing network topologies having fractional dimensions greater than 3. Thus it seems brains have both fractal geometry and fractal topology, although how these two aspects of brain organization are related to each other is a fascinating question we will leave unaddressed for the moment.

In VLSIs, such high dimensional interconnect topology is related to logical capacity of the circuits, and we suggest that it is also likely to be functionally advantageous in nervous systems. However, greater than three-dimensional connection topology incurs an extra wiring cost, compared to the minimum cost of wiring the same set of physically located nodes interconnected by a nearest-neighbour, lattice-like topology with 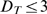. Our results indicate that nervous systems and computational circuits are cost-efficiently but not cost-minimally embedded in physical space, meaning the wiring length of these networks is close to the minimum length it could be, given their high dimensional topology, but it is not absolutely minimized.

Previous studies of brain and neuronal networks have shown that wiring costs are nearly if not absolutely minimized [15], [42], using a combination of component placement optimization and wiring placement optimization. Component placement optimization creates minimally wired networks by retaining the connectivity of the system (edges) while allowing components of the systems (nodes) to move in space. This approach maintains the inherent functionality of the system and asks whether, given such functionality, the components can be ordered in a different way to provide shorter average wiring. Conversely, in wiring placement optimization [15], we retain the placement of the components of the system (nodes) and alter the connectivity of the system (edges). As such, wiring placement optimization does not retain the inherent functionality of the systems but instead retains the inherent anatomical structure (heterogeneous localization of brain regions or neurons). In this work, given the placement of nodes in space and preserving the number of edges, we ask: could these nodes be reconnected in a different configuration so as to yield a shorter average wiring length? Our purpose in choosing wiring placement over component placement was to compare the topological and physical characteristics of a given brain network to those of a 3-dimensional lattice-like network in a realistic brain anatomy. By this approach we found that wiring costs in brain networks were nearly but not absolutely minimal.

Given the high metabolic costs of the brain (about 20% of the total energy budget for only 2% of body mass in the human), of which a large proportion is due to the costs of building and maintaining functional connections between anatomically distributed neurons [43], [44], it seems reasonable to ask: why have brain wiring costs not been *more* strictly minimized by natural selection? Our answer to this question is that the selection of greater than 3-dimensional (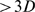) network topologies, which are associated with hierarchical modularity and greater logical capacity, has been prioritized despite the adverse impact on wiring cost that is entailed when any system that is topologically more complex than a 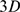 lattice is embedded in physical space [15], [42]. Absolute minimization of wiring cost in these nervous systems could only be achieved at the expense of reduced topological complexity. Moreover, the generalizability of this result to both *C. elegans* and *Homo sapiens* suggests that a trade-off between high dimensional connectivity and wiring cost has been negotiated in the evolution of nervous systems at microscopic (cellular) and macroscopic (whole brain) levels of description and in phylogenetically removed species.

### Rentian and allometric scaling of nervous systems

In comparing the results of these and other studies, it is important again to highlight the distinction between Rentian characteristics for partitioning (topological Rentian scaling) versus placement (physical Rentian scaling) [23]. Partitioning examines the un-embedded network topology, whereas placement examines the position of nodes embedded in a physical substrate. Therefore, the partition-based Rent exponent measures a characteristic more intrinsic to the VLSI circuit topology while the placement-based Rent exponent measures a characteristic of the extrinsic physical wiring properties [23]. In the construction of a VLSI, placement is the artificial process by which a given network topology is somehow embedded into a physical substrate by the manipulation of nodal placement. Minimization of wiring costs is an important economic factor in VLSI production and designers will seek to optimise the efficiency of network embedding. The optimal cost-efficient embedding will have a physical Rent exponent, based on placement, equivalent to the topological Rent exponent, based on partitioning. However, optimal placement is an NP-complete problem, as is optimal partitioning, and as such different placement algorithms can yield sub-optimal Rent exponents.

While topological Rentian scaling of the *C. elegans* connectome has been previously reported [45]–[47], the present work is the first, to the best of our knowledge, to report topological Rentian scaling in human anatomical networks derived from neuroimaging and also the first to explore physical Rentian scaling in neuronal networks. In the nervous systems studied here, the placement and topology have both been evolved by nature, and as such the physical Rent exponent is constrained by the (sub)optimality of natural selection rather than by the particular placement algorithm chosen (as is the case for a VLSI). We found that for both human brains and the nematode connectome, the physical Rent exponent 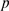 was close to its theoretical minimum, the topological exponent 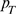, indicating that natural selection has resulted in near-optimisation of cost-efficient network placement.

This analysis has also provided the first direct evidence for a simple relationship between physical Rentian scaling of connectivity within the nervous system of a single species and allometric scaling of gray and white matter volumes across the differently sized brains of a range of mammalian species. This result needs to be considered in the context of a rich prior literature on allometric brain scaling and its possible theoretical relationship to isometric or fractal scaling of network connectivity. Early studies of allometric scaling in the brain showed, for example, that the number of neurons scaled with gray matter density while the number of synapses remained constant [48]–[50]. The study of the relationship between white and gray matter in mammalian cortex began with the work of Schlenska [51] and later Frahm [52] in the late 70s and early 80s. Both reported scaling relationships in independent mammalian datasets with exponents of 1.22 and 1.24 respectively. The strength and consistency of this finding, later underscored by a comparative MRI study [53], prompted theoreticians to propose various geometric and mechanistic models which have been used to predict other scaling relationships between related neuronal variables [54], [55], serving as important guides for further experimental work.

Beiu et al. suggested that the allometric scaling exponent for gray and white matter volumes between species was *identical* to the Rent exponent within a species [56]. The assumption that the scaling exponent between white and gray matter volumes is identical to Rent's exponent necessarily neglects the differences between a volumetric scaling and a network scaling (e.g., a scaling of nodes and connections). As we have shown in our derivation here and in the supplementary Text S1, nodes and connections do not in fact scale directly with volume, and thus the exponents of volumetric scaling and Rentian scaling are not directly equivalent.

Prothero developed a repeating units model [54] which suggests that all brains are made up of identical repeating units: larger brains simply have more of these units than smaller brains. Changizi developed a slightly more complicated, two-part model [55] partially based on the application of West's theory of branching to neuronal arbors [57]. While these two models sought to describe allometric relationships between a wide variety of neuronal variables, Zhang and Sejnowski elegantly propounded a model to explain only the allometric relationship between gray and white matter [25]. However, following the publication of these models, empirical developments have challenged one of the main assumptions underlying all three: namely, that there is a basic uniformity of the cerebral cortex as evidenced by a constant number of neurons in a unit area of cortical surface; this assumption now seems unrealistic [58]. Also, although the final two models produce an near-perfect 4/3 scaling exponent between white matter volume and cortical gray matter volume in mammals, they do not readily allow for distinct scaling exponents in non-cortical systems, e.g., cerebellum, or in non-mammalian species.

These challenges aside, prior studies have contributed seminal insights to our understanding of allometric scaling of brain properties which we hope we have been able to further refine. We suggest that the allometric scaling of white matter volume with gray matter volume is a direct consequence of the physical Rentian scaling of connectivity in a given brain. In contrast to the models previously described, our explanation allows an independent empirical validation or cross-check: we separately estimate the Rentian scaling within a single mammalian system and use it to predict the allometric scaling of white matter volume with gray matter volume across a range of mammalian species. In addition, our heuristic allows for differences in scaling relationships between distinct areas of cortex such as the neocortex and cerebellum or between different classes of animals such as vertebrates and invertebrates. This isometric generative mechanism for allometric scaling does not stand or fall by producing an ideal, e.g., 4/3, scaling relationship between white matter volume and gray matter volume, but allows for irrational or non-integer scaling exponents that may vary somewhat depending on the type of brain network and/or the phylogenetic class of species considered.

However, like the other available models, our derivation does include some assumptions or approximations: 1) we have made the approximation of ignoring the effect of white matter dilation after determining that its contribution is small over the range of white matter volume values studied (see supplementary Text S1), and 2) we have assumed that the number of synapses in a cross-sectional area is constant as a function of gray matter volume based on the known invariance of synaptic density [50]. Empirically required alterations to these approximations and assumptions may induce small corrections to the estimation of the Rent exponent, 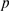.

The formal and empirical connection we have made between fractal or self-similar connectivity of the nervous system of a single mammalian species and the allometric scaling of gray and white matter volumes over multiple mammalian species provides a novel mechanistic explanation for a long-established observation. We propose that allometric scaling of brain anatomy is constrained by fractal properties of the cortical network for information transfer in broadly the same way as the allometric scaling of mammalian metabolic rate with body mass is constrained by fractal properties of the respiratory network for gas exchange [57], [59].

### Methodological limitations

There are several methodological issues to be considered in evaluating the results of this study. The small size of both the human MRI and *C. elegans* networks limits the precision with which we have been able to estimate the fractal dimension of network topology, 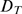. We have tried to address this by reporting convergent results from two complementary estimators (topological Rentian scaling and box counting) and by using DSI data which have been parcellated into 1000 nodes. Nonetheless in future studies, it will be useful to apply finer grained parcellation templates to human neuroimaging data to improve estimation of fractal properties of network topology by analysing the systems over a larger range of scales.

The use of covariation in gray matter volumetric variables as a measure of anatomical connectivity between brain regions [12], [60]–[62] is indirect and entails some assumptions. For example, it has been assumed that reciprocal afferent connections have a mutually trophic effect on the growth and maintenance of both connected regions leading to positively correlated volumes in adult brains [13], [62]. Recent studies have provided some experimental validation of this hypothesis by comparing pairs of regions with highly correlated volumetric properties to known fiber tracts established using diffusion tensor imaging [13], [63]–[65] and tract tracing studies [66]–[68]. Nevertheless, the assertion of anatomical connectivity on the basis of regional covariation in gray matter volumes remains somewhat conjectural at this time. Moreover, the construction of a single group mean anatomical network from the MRI data means that the error in estimation of the Rent exponent 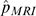, may be under-estimated by exclusion of any between-subject or between-network components of variability.

The diffusion spectrum imaging network, on the other hand, contains an inherent distance bias [14], meaning that long distance connections have a lower probability of being included in the network than short distance connections. While Hagmann and colleagues did use a distance bias correction in the preprocessing of these networks, the most complete correction method remains a matter of ongoing debate [14]. It is possible that some distance bias remains in the current dataset which may artefactually decrease the topological dimension, 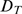, the Rent exponent, 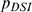, and the average wiring length, 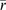. However, it is not the purpose of this study to evaluate the available methods for distance bias correction and we have instead used this recently published dataset which represents one of the currently accepted methods.

This previously published DSI dataset [14] includes data for 5 subjects with 1 subject scanned twice. As such, this dataset is not adequate to assess the inter-scan reliability or inter-subject reliability of the anatomical structural properties we are studying in this work. Recently, it has been shown that similar whole-brain networks derived from functional MEG data have reproducible topological properties [69]. However, a similar study in anatomical networks has not yet been published, and it will be important in future work to describe the reproducibility of network architecture in terms of both topology and physical embedding.

In this work, the distance between any two network nodes was defined as the Euclidean distance between the center of mass of the brain regions (in the human) or neuronal cell bodies (in *C. elegans*). While this definition is currently widely used [11], [15], [63], [70], it provides an indirect estimate and likely an under-estimate of the true length of white matter tracts and axons in these neural systems, which may take convoluted paths to connect a given pair of nodes. Future advances in diffusion imaging may provide us with better length estimates for major white matter tracts in the the human brain while advances in the microscopic characterization of neuronal tissue may provide us with better estimates of individual axonal pathways.

The placement embedding for the VLSI circuit required the use of simulated annealing. The estimated physical Rent exponent based on placement, 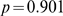, was less optimal than a previously reported Rent's exponent based on partitioning [71]. It is important to be aware that Rent exponents based on placement and based on partitioning may not be identical; the partitioning method does not require simultaneous physical embedding of all gates in the entire system. We have chosen to use the placement embedding technique to make the results most comparable to the *C. elegans* and human brain network results. In a similar vein, it is important to note that we used the formalism of graphs and edges rather than hypergraphs and hyperedges. The latter are often used in the analysis of VLSI circuits but the concepts are not simply transferable to the biological networks studied here. Thus we have chosen to use simple edges in all reported analyzes to facilitate comparability across systems.

The relationship between allometric scaling and Rentian scaling could be further supported by studying Rentian scaling in MRI or DSI/DTI datasets from a range of mammalian species rather than the human alone. In particular, it would be interesting to discern whether there is a difference in isometric Rentian scaling between mammalian and non-mammalian species as well as between marine and terrestrial mammals who arguably show distinct volumetric scaling relationships [72], [73]. The construction of a comparable species-dependent MRI network would require structural scans from over 200 animals in that species. While no such data is currently available or likely to become available in the near future, the application of DTI specifically to the macaque monkey is a pressing line of current inquiry.

### Conclusion

The parallels we have identified between the properties of naturally selected nervous systems and commercially selected computational systems suggest that diverse information processing networks have convergently evolved to satisfy ubiquitous fitness criteria. Just as principles of natural selection were originally informed by Darwin's analysis of artificial selection pressures operating in the market for domestic animals, principles of nervous system evolution may be elucidated by comparative analysis of computational systems that have evolved in the market for logically advanced computers.

## Materials and Methods

### Network data

For the *C. elegans* nervous system, connection data and two-dimensional spatial coordinates for each neuron were taken from [15], [16]. Each neuron was taken to be a node in the network and nodes were connected by edges where a chemical or electrical (gap junction) synapse between two neurons was known to exist. For the human nervous system, we used two sets of neuroimaging data from independent samples studied using complementary magnetic resonance imaging (MRI) methods; these include the most fine-grained view to date of whole brain white matter tract connectivity and the classical cytoarchitecturally constrained view of whole brain gray matter connectivity. It was hoped that in the combination of both complementary lines of inquiry, the discovery of consistent properties would underscore both replicability and robustness. In 259 healthy adults, regional gray matter volume measurements were made in 104 cortical and subcortical regions defined by an anatomically informed parcellation template applied to conventional MRI data [11]. The inter-regional partial correlation in gray matter volume was estimated for each pair of regions and thresholded to create an undirected graph where each node corresponded to a region and an edge indicated a suprathreshold correlation of volumes between regions [11], [13], [63]. In 5 healthy adults with 1 adult scanned twice, the probabilities of fiber tracts between any two regions of interest (N = 1000) were determined from diffusion spectrum imaging (DSI) data using an altered path integration method [14]. The connectivity backbone of this probability matrix was determined by first calculating the minimum spanning tree and then adding connections with the highest probability weights until the average degree was 4 [14]. For the VLSI (s953) circuit [17], each node in an undirected graph represented one of 440 logic gates and an edge represented a wire between gates. The *C. elegans* data is freely downloadable from the Biological Networks website http://www.biological-networks.org/; the DSI data are freely downloadable from the Brain Connectivity Toolbox www.brain-connectivity-toolbox.net.

### Hierarchical modularity

To visually represent the hierarchical community structure of the networks, we used a co-classification algorithm which iteratively determines hierarchical nodal affinities based on topological overlap in the symmetrized matrix and uses this information to determine the relative relationships between nodes at all hierarchical levels [74]; see Figure 1. Modularity of these matrices was estimated using the Louvain community detection algorithm [18] and compared to the modularity distribution (N = 100) of two benchmark networks: 1) Pure random networks, i.e., networks with the same number of nodes and edges as the original network, and 2) Functional random networks, i.e., those with the same number of nodes and degree distribution as the original networks [75] such that each edge was rewired on average 15 times. A network was defined as being hierarchically modular if it contained first-level modules with significantly non-random modularity, i.e., the presence of submodules was confirmed; see supplementary Text S1 for details.

### Topological dimension

The fractal dimension of the network topology 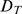 was estimated in two ways. First, we used a topological partitioning algorithm (hMetis software, version 1.5) to compute the topological Rent's exponent, 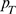, which is then related to the topological dimension by 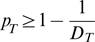 [5]. The network was recursively partitioned into halves, quarters, and so on in topogical space. The slope in log-log space of the average number of nodes in a partition versus the average number of edges crossing the boundary of the topological partition was defined as the topological Rent's exponent, 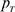. In a complementary analysis, we estimated 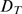 using the box-counting algorithm of Concas et. al [21] based on Song's renormalization algorithm [22]. This estimator counts the number of boxes 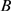 required to cover all nodes in each network as box size 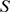 is varied between 1 and 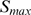. The gradient of a straight line fitted to 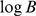 versus 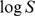 using weighted linear regression is a consistent estimator of 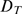; see Figure 1.

### Average expected wiring

The expected wiring of a high dimensional topology which is embedded in a lower dimensional physical space is
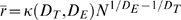
(4)for 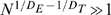, where 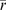 is the mean connection distance in terms of node-to-node spacing, 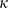 is a constant of order unity, 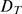 is the fractal dimension of the topology, 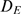 is the Euclidean dimension of the embedding, and 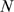 is the number of nodes [5], [24]. The node-to-node spacing, 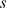, is given by
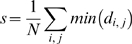
(5)where 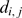 is the Euclidean distance between any pair of nodes 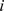 and 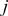. The mean connection distance in terms of node-to-node spacing, 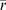, is then given by
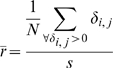
(6)where 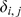 is the Euclidean distance between any pair of *connected* nodes 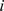 and 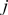.

### Rentian scaling in Euclidean space

The Euclidean space of the networks was tiled with 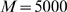 overlapping randomly sized boxes (e.g., two-dimensional squares or three-dimension cubes for the VLSI, *C. elegans*, and human networks. In each box we determined the number of nodes (*n*) and the number of connections (*e*) that cross the box boundaries; see Figure 1. The gradient of a straight line fitted to 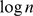 versus 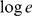 using iteratively weighted least squares regression is an estimate of the Rent exponent 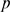; see Figure 3. To minimize (Region II) boundary effects, 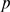 was estimated using the subset of boxes which contained less than half the total number of nodes, 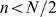.

### Minimally and randomly wired networks

Each network was minimally rewired by first computing a minimum spanning tree to ensure that all nodes were connected then iteratively adding the next shortest edge to the network until the connection density matched that of the observed networks [15]; see Table 1. Randomly wired networks were pure random networks with the same number of nodes and edges as the observed network; see Table 1, bottom panel.

### Allometric scaling

If we approximate the brain as a sphere, then the cross-sectional area, 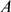, of white matter volume, 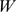, is given by
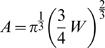
(7)which can be related to the number of connections, 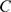, according to
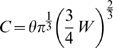
(8)where 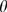 denotes the number of connections per unit surface area of white matter which we assume to be constant given the independence of synaptic density and brain volume over the mammalian class of species [50]. We use 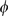 to denote the number of constant-complexity processing elements, 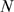, per unit volume of gray matter, 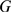, which scales with synaptic density and is therefore a constant. On this basis, we can write
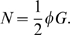
(9)A system obeys Rent's rule if 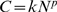 for some Rent coefficient 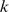 and exponent 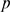; inserting (8) and (9) we then have:
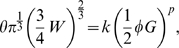
(10)which simplifies to
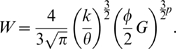
(11)Thus,
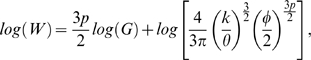
(12)and multiplying the allometric scaling exponent 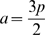 by 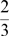 provides an estimate of the Rent exponent, 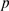. For a more detailed derivation, see supplementary Text S1.

## Supporting Information

Text S1 Supplementary Information
(1.22 MB PDF)
