# Supplementary material for: Efficient Physical Embedding of Topologically Complex Information Processing Networks in Brains and Computer Circuits
Source: PLoS Comput Biol. 2010 Apr 22;6(4):e1000748. doi: 10.1371/journal.pcbi.1000748 (PMC2858671; doi:10.1371/journal.pcbi.1000748)
Supplement: Text S1 — Supplementary Information (1.22 MB PDF) [file pcbi.1000748.s001.pdf]

Efficient physical embedding of topologically complex information processing networks in brains and computer circuits

SUPPLEMENTARY TEXT S1

Danielle S. Bassett[1,2,3,6,†], Daniel L. Greenfield[4,†], Andreas Meyer-Lindenberg[5], Daniel R. Weinberger[6], Simon W. Moore[4], Edward T. Bullmore[3,*]

1 Department of Physics, University of California Santa Barbara, Santa Barbara, CA, USA

2 Institute for Collaborative Biotechnologies, University of California Santa Barbara, USA

3 Behavioral & Clinical Neurosciences Institute, University of Cambridge, Department of Psychiatry, Herchel Smith Building for Brain & Mind Sciences, Cambridge UK

4 Computer Laboratory, University of Cambridge, Cambridge, UK

5 Central Institute of Mental Health, Mannheim, Germany

6 Genes, Cognition, and Psychosis Program, Clinical Brain Disorders Branch, National Institute of Mental Health, Bethesda, MD, USA

† These authors contributed equally.

∗ E-mail: Corresponding etb23@cam.ac.uk

Contents

1. Quantification of Hierarchical Modularity
2. Estimation of the Topological Dimension
3. Topological Rentian Scaling versus Box-Counting
4. Estimation of Rent's Exponent
5. Detailed Version of Allometric Scaling Derivation
6. Description of Methodological Limitations
7. Figure 1, "Full hierarchical modularity decomposition for the *C. elegans* neuronal network"
8. Figure 2, "Full hierarchical modularity decomposition for the human anatomical network as measured by DSI"
9. Figure 3, "Topological Rentian scaling in information processing networks"
10. Figure 4, "Fractal dimension of the observed and minimally wired networks"
11. Figure 5, "Goodness of fit in modeling the Rentian relationship"
12. Figure 6, "Dependence of topology on addition of random and H-tree links"
13. Figure 7, "Error associated with the number of partitions and random sampling"
14. Figure 8, "Rentian characteristics of minimally wired networks"
15. Table 1, "Hierarchical modularity in computational and nervous systems"
16. Table 2, "Hierarchical modularity in DSI data"
17. Table 3, "Topological Rentian scaling and fractal topology"
18. Table 4, "Individual measures of physical embedding"

Quantification of Hierarchical Modularity A network was defined as being hierarchically modular if it contained first-level modules with significantly non-random modularity, e.g., first-level modules contained submodules. To visually represent the hierarchical community structure of the networks, we used a co-classification algorithm which iteratively determines hierarchical nodal affinities based on topological overlap in the symmetrized matrix and uses this information to determine the relative relationships between nodes at all hierarchical levels [1]; see Figure 1 and 2 in the main text. The modularity of these matrices was estimated using the Louvain community detection algorithm [2] and compared to the modularity distributions (N=100) of two benchmark networks: 1) Pure random networks, i.e., networks with the same number of nodes and edges as the original network, and 2) Functional random networks, i.e., those with the same number of nodes and degree distribution as the original networks [3] such that each edge was rewired on average 15 times. All four network types were found to have modular structure larger than expected in random networks; see Table 1, top panel.

The topological structure of each network was further characterized by 1) the number of levels found in the Louvain decomposition, 2) the number of modules in the first level decomposition and 3) the average modularity of the first-level modules; see Figures 1 and 2 and Tables 1 and 2 in this supplementary text in addition to Figure 2 in the main text. We found that the number of hierarchical levels defined by the Louvain decomposition was not significantly different than pure random or functional random networks, suggesting that this quantity is largely dictated by the number of nodes and number of edges in the original system; see Table 1, Panel 2. The number of modules in the first level decomposition did not show any particular relationship to the same quantity in either of the random network types; see Table 1, Panel 3. The average modularity of the first level modules was found to be significantly higher than expected in random networks in both the *C. elegans* neural network and the human brain networks derived from DSI data; see Table 1, bottom panel. While the *average* modularity of the first-level modules of the VLSI network and the human brain network derived from MRI data was not different than expected in a random network, several modules in both networks showed significantly non-random submodular structure. Figure 2 in the main text shows the hierarchically modular structure of all four network types in detail. In each of these figures, we first show the first level modules estimated by the Louvain decomposition. For each module separately, we estimate the modularity. We then iteratively rewire the module 100 times to create a distribution of functional random modules of the same size and with the same degree distribution as the original module. We estimate the modularity of these functionally random

modules as well as the modularity of 100 purely random networks that are the same size (same number of nodes and edges) as the original module. We perform a 1-tailed t-test to determine if the modularity of the original module is larger than expected in the distribution of 100 functional random networks, and a second 1-tailed t-test to determine if the modularity of the original module is larger than expected in the distribution of 100 pure random networks. In the figures, we report the p-values for these two tests below the modularity value of each module. By this process of iterative coarse-graining and rewiring, we are able to characterize the modular, submodular, and in some cases deeper hierarchical structure of the networks.

Visual inspection of the hierarchical structure of the 4 types of networks from information and nervous systems (Figure 2 in the main text) suggests that they each decompose slightly differently. The VLSI network decomposes into many first-level modules, only one of which leads to a tree 4 levels deep. Like the VLSI network, the human brain MRI network shows hierarchical structure descending from its medium-sized modules rather than its largest module. Contrarily, throughout the decomposition of the *C. elegans* network, there seems to be a direct relationship between the size of a module and the depth of its hierarchical substructure. The human brain DSI network is the most homogeneous of all, in which each level is composed of $\sim 2-3$ subcomponents of similar size. Further work is necessary to determine whether or not these features are characteristic of each type of network and therefore can be used to distinguish between them.

Estimation of Topological Dimension We first estimated the topological dimension of all four types of information processing networks by computing the topological Rent exponent, p_T, which measures the configuration of the network in topological rather than physical space [4]. The topological Rent exponent, p_T, is related to the minimal (intrinsic) topological Rent exponent, p_{min}, by $p_T \geq p_{min}$. Since p_{min} is related to the topological dimension of the network (by $p_{min} = 1 - \frac{1}{D_T}$ [5]), we can relate the topological Rent exponent to the topological dimension of the network: $p_T \geq 1 - \frac{1}{D_T}$.

Recently, Partzsch et al. examined the topological network of *C. elegans* and showed that its topological Rent exponent was high [6]. The estimation method used was a type of spectral partitioning algorithm which, at its inception in 1994, was suggested to provide "the lowest possible p [Rent's exponent]". Since that time, more advanced simulated annealing approaches have been developed such as in the software hMetis [7] which was used in another recent study of Rentian scaling in biological systems by

Reda [4]. Interestingly, the topological Rent exponent for *C. elegans* reported by Reda [4] is lower than that reported by Partzsch et al. [6] and consistent with both the physical and topological Rent exponents reported in the present work.

Given this methodological history, we computed the topological Rent's exponent using the advanced simulated annealing approach in the software program hMetis version 1.5; results can be seen in Figure 3. In this method, the network is recursively partitioned into halves, quarters, and so on in topological space. The slope in log-log space of the number of nodes in a partition versus the number of edges crossing the boundary of the topological partition was defined as the topological Rent's exponent, p_T. For all of the networks, these log-log plots demonstrated a clear Region I (linear scaling indicating fractal topology) and Region II (for high partition sizes, where the curve begins to fall like a shepherd's hook, indicating topological boundary effects). The presence of topological scaling in Region I indicates that the networks contain a fractal topology. The slope of the line (measured using weighted linear regression) through the points in Region I is the topological Rent's exponent, p_T, which was then used to estimate the dimension of this fractal topology, D_T; see Table S3.

Note: The terms "Region I" and "Region II" are from the VLSI community, and denote where internal connectivity dominates and where external connectivity starts to dominate respectively. For example, at the right-most side of the VLSI chip, there are no further nodes to connect to on the right, and instead there is a much smaller number of expensive connections going off-chip. Thus if a partition covers the right-most side of the chip, the total count of 'terminals' gets markedly reduced. A similar effect holds topologically. At large scales this effect dominates and accounts for the Region II drop-off in terminal count.

Since each of the networks portrayed topological Rentian scaling consistent with a fractal topology, we also directly estimated the fractal dimension of the network topology, D_T, using the box counting algorithm of Concas et. al [8] (see following section for a theoretical comparison of these two methods). This estimator counts the number of boxes B required to cover all nodes in each network as box size S is varied between 1 and S_{max}. The gradient of a straight line fitted to $\log B$ versus $\log S$ using weighted linear regression is an estimate of D_T whose error decreases as the size of the system increases; see Figure 4. Resulting estimates for D_T are consistent with estimates from the topological Rentian scaling method described in the previous paragraph; see Table S3. Despite this consistency, a limitation of the current work is that both estimations were performed on a few data points and scaling was present over less than

a single order of magnitude; therefore results must be interpreted carefully.

As a third method of estimation, we have implemented the renormalization scheme suggested by Song et al. in [9], which coarse-grains the system into boxes of a given size. This method, originally applied to networks of several hundred thousand nodes, works by grouping nodes into boxes of different sizes, and therefore should intuitively show fewer levels or scales within the network than the pure box counting technique. Indeed, we found that after applying the renormalization scheme, all systems have many fewer points. The two end points are trivial; there is one box covering all the nodes in the network and there are N boxes of size 1. Across all four systems, only 1 or at most 2 “middle” points are available for use in computing the fractal properties, which is far less than the number of points given by the method of Concas et. al [8].

Topological Rentian Scaling versus Box-Counting There are important differences in fractal behaviour for Rentian scaling and Box-Counting measures. Significantly, Rentian scaling is fully compatible with small-world networks, whereas Box-Counting is not. As many networks including neuronal ones are likely to be small-world, it is important to elaborate on their differences.

As described above, we estimated the topological dimension of the information processing networks using both topological Rent exponents and box-counting. We observed that fractal scaling was clearly visible in the Rentian anaylsis and less visible in the box-counting plots. It is known that small-world properties affect the scaling measured by a box-counting analysis [10]. Here we compare the effects of small-world properties on the estimation of the topological dimension of networks using either box-counting or topological Rentian analysis.

Work by Rozenfeld [10] has shown that there is some compatibility between fractal box-counting dimension and small-world topologies. However, even though the fractal topology of a network might be seen at small scales, the small world nature means that there is a single box that covers the entire network with side-length of order $\log N$. This means that the small-world behaviour rapidly dominates in box-counting, leading to an exponential cut-off of the power-law behaviour.

Compared to box-counting, Rentian scaling looks at how the number of edges crossing a boundary scales with the number of nodes inside it. In VLSI, Ozatkas has observed that this can be thought of as relating the volume of logic inside the boundary, to the flow of information (surface area) across that boundary in terms of edges [5]. The Rent exponent is then given by the ratio of surface area scaling to

volume scaling - and can thus be related to the dimensionality of information flow scaling. One can easily show that large d-dimensional meshes have Rent exponents of $p = (d-1)/d$. Moreover, we can define the Rentian information-flow dimension to be:

$$d = 1/(1-p)$$

Whereas the topological distance used for assessing small-world behaviour and in box-counting analyses examines the number of hops from one node to another, it isn't concerned with the quantity of information flow between them. A single link may connect two small world sub-networks, and thus allow only a small flow of information between these two sub-networks whilst maintaining small-world behaviour. That is, the single link is shared between all the nodes on one sub-network that want to communicate with a node on the other sub-network. Although such a network may be small-world, one has to wonder whether that single link is sufficient for all the information flows required for solving real computational problems - certainly despite the desire to reduce wiring costs, such connectivity patterns generally aren't practical in VLSI. A Rentian analysis, however, is primarily concerned with the quantity and scaling of communication required, rather than topological distance.

VLSI networks are known to obey both fractal Rentian behaviour and small-world topologies [11,12]. We should emphasise that these are mutually compatible attributes. For VLSI, a clock tree, typically an H-tree [13], uniformly distributes clock timing information to disparate parts of the logic network, so that they can synchronise their communication. H-trees are called thus because their basic structure consists of physical links, in the 2D space of a VLSI chip, that look like the letter H. At each of the four ends of the H, another H of half the size is attached at its middle, and so on, thus forming a fractal tree of H's. Clock trees are very expensive from both a manufacturing and power-consumption standpoint. The H-tree emerged as a way to achieve the goal of reducing communication skew at leaf nodes (ensuring that the logic is globally synchronized as a small-world), whilst keeping costs reasonable. We should point out that clock trees are by no means the only cause for small-world topologies, and that the rest of the circuitry may also exhibit small-world behaviour for other reasons.

In Figure 5A we see the effect on network-diameter of adding multiple levels of an H-tree versus adding random links to a 2-D mesh (not torus), resulting in a small-world network for both types. Here, each "H" of the H-tree consists of four links to a central node. In Figure 5B we also see that the Rentian

behaviour and exponent are only very slightly affected by the addition of an H-tree, whereas the random links lead to a large divergence in the Rentian behaviour and exponent. Thus the *nature* of the small-world behaviour is important in assessing its impact on Rentian scaling. In the case of random links, the number of links cut by partitioning approximately grows by the number of nodes in each partition, whereas in the case of the H-tree, the number of links cut is small and approximately constant at each level of partitioning. This compares with the 2-D mesh, where the number of links cut grows approximately by the square-root of the number of nodes in each partition. Importantly, unlike the box-counting metric of Song [9], where small-world properties dominate over any fractal connectivity, the small-world property does not necessarily impact on the physical and topological measures of Rentian fractal behaviour.

In VLSI networks, the Rentian behaviour and homogeneity of embedding results in an approximately power-law distribution of physical wiring distances [12], and is used in roadmaps for costs, manufacturing processes, design and prediction of future processor performance [14]. It is important to distinguish between the resultant power-law nature of physical link distances versus the potentially non-power-law nature of topological distances, especially in small-world networks.

Estimation of Rent's Exponent While visual inspection of n versus e plots in log-log space suggest that the two are related to each other by a power-law, it is important to quantify the goodness of fit of the power-law model in comparison to other possible models. We therefore tested a variety of fits to the relationship between the number of nodes in a physical partition, n, and the number of edges crossing the boundary of the partition, e. Using a nonlinear least squares regression, we modeled a power-law, exponential, logarithmic, linear, quadratic, cubic, and higher order polynomials; each model contained two free parameters, C and α, and therefore their respective errors were directly comparable. For each fit, we determined the root mean squared error (*rms*) of the fit; see Figure 6 for *rms* error for each fit over all of the information processing systems. In all networks, the power-law fit provided the least error, suggesting that a power-law is a good estimate of the relationship between the two variables, n, and e.

Further, there are two sources of possible error in the estimation of Rent's exponent, p. First, there is the error in the fit of the power-law to the n versus e plot in log-log space; these errors are, for example, given in Table 1 of the main text. Secondly, there is an error associated with data sampling. Each data point represents a physical partition of the system (in which we count n nodes and e edges crossing the boundaries of the partition). In the current work, we partitioned the system 5000 times (i.e., constructed

5000 random partitions) and fit the power-law model to these 5000 observations. However, we could randomly partition the system 5000 more times, and fit the power-law model to these 5000 observations and maybe get a slightly different estimate of p than in the first partitioning. Therefore, in addition to quantifying the error associated with the fit, we have also quantified the error associated with the number of observations (partitions) used to construct the fit as well as the error associated with the random nature of the sampling; see Figure 7. As is perhaps expected, the error due to resampling decreases as the number of observations per fit increases. Importantly, however, the average estimated Rent's exponent remains relatively independent of the number of observations used to estimate the power-law fit.

Detailed Version of Allometric Scaling Derivation In order to derive a relationship between allometric and Rentian scaling exponents in the brain, we need to define (1) the number of connections in a cross-sectional area as a function of the white matter volume, and (2) the number of processing elements in the whole system as a function of the gray matter volume.

In VLSIs, the wiring of the circuit is located in multiple layers above the logic of the circuit. Thus, the area of the logic-limited circuit is equal to the area of the logic, $V = G$. In the human brain, however, the white matter tracts are embedded in the same space as the "logic" or gray matter. The "wiring" of the white matter tracts causes the "logic" or gray matter segments to be farther apart from each other. Thus, the volume of the system is given as $V = W + G$, which is an increase on G of $(W + G)/G$ or $(1+W/G)$. This stretching further causes an increase in the white matter volume, W, by increasing axon length. Since axon length is a one dimensional linear term, the dilated white matter is larger than the undilated white matter by a stretching constant μ given by the cube root of the total increase:

$$\mu = \sqrt[3]{1 + W/G}. \tag{1}$$

Over the scale of the mammalian white matter, this dilation effect is very small. Even if W varies as widely as $W \sim (0, G]$, μ will vary as $\mu \sim (1, 1.26]$. In $\log_{10}$ space, this variation in μ becomes $\log_{10}(\mu) \sim (0, 0.1]$ which is a small perturbation of $\log(W)$, over the range of mammalian white matter values. Thus, it is possible to approximate the dilated white matter volume $W_{dilated}$ by the undilated white matter volume $W_{undilated}$.

$$W_{dilated} \sim W_{undilated} \tag{2}$$

In the remainder of this derivation, the simple symbol W will be used to refer to $W_{dilated} \sim W_{undilated}$.

To determine the number of connections in the cross-sectional area, S, as a function of the white matter volume, W, we first approximate the brain as a sphere and therefore the cross-sectional area, A, of the brain as equivalent to the area of a circle:

$$A = \pi r^2, \tag{3}$$

where r is the radius of the sphere, which can be rewritten as

$$A = \pi^{1/3}\pi^{2/3}r^2. \tag{4}$$

We also know that the volume, W, of the sphere is given by

$$W = \frac{4}{3}\pi r^2, \tag{5}$$

which can be rewritten as

$$\frac{3}{4}W = (\pi r^3)^{2/3} = \pi^{2/3}r^2. \tag{6}$$

Note that the right hand side of Eq 6 is also found in Eq 4. Substituting, we find that

$$A = \pi^{1/3}(\pi^{2/3}r^2) = \pi^{1/3}(\frac{3}{4}W)^{2/3} = C_1 W^{2/3}, \tag{7}$$

where $C_1 = \pi^{1/3}(\frac{3}{4})^{2/3}$ contains all constants independent of W.

It is important to note that while we suggest that white matter volume can be approximated by a sphere, any other 3-dimensional volume will work equally well, as has been previously pointed out by Prothero [15]. The cross-sectional area of any 3-dimensional object will scale as 2/3 the volume of that object. Shapes other than a sphere will change the constant, C_1, but will leave the exponent, 2/3, unchanged.

By definition, the number of connections within the cross-sectional area can be written as

$$S = \theta A, \tag{8}$$

where S is the number of synapses, θ is the number of synapses per unit area, and A is the cross-sectional area. From prior anatomical data (see [16]), we find that the number of synapses per unit volume is independent of white matter volume, W, and therefore the number of synapses per unit area, θ, is also independent of W. So we can now define θ to be

$$\theta = C_2. \tag{9}$$

Substituting Eqs 7 and 9 into Eq 8, we can write the number of synaptic connections S as a function of white matter volume, W:

$$S = \theta A = C_1 C_2 W^{2/3} = C_3 W^{2/3}. \tag{10}$$

where $C_3 = C_1 C_2 = \pi^{1/3}(\frac{3}{4})^{2/3}\theta$.

To define a relationship between gray matter volume G and the number of processing elements N, we first recall that the number of neurons in the brain scales disproportionately slowly with the gray matter volume as $G^{2/3}$ while the number of synapses scales directly with G [16]. Therefore, the number of synapses per neuron, or synaptic complexity of the neurons, is increasing with brain volume. In computer circuits, we also have gates that vary in complexity, and circuits can even be built up of macrocells that have very high complexity. However, to perform the gate count needed for Rentian analysis in VLSIs, we must count computing elements in comparable terms, typically of the simple 2-input logic gate (NAND2). Thus, larger and more complex computing elements are counted as multiple NAND2 gates. In order to work with the same counting statistics in the human brain, we define a constant-complexity processing unit as using a fixed number of synapses. Since the number of synapses scales with G, then so does the number of constant-complexity computing units in the gray matter:

$$N = \phi G, \tag{11}$$

where ϕ is the number of constant-complexity computing elements per unit volume.

Now we can rewrite the Rentian scaling relationship

$$S = kN^p, \tag{12}$$

where k is the Rent coefficient and p is the Rent exponent, in terms of white matter W and gray matter G volumes:

$$C_3 W^{2/3} = k(\phi G)^p, \tag{13}$$

or

$$W = C_4 G^{3p/2}, \tag{14}$$

where C_4 is $\frac{k\phi^p}{C_3} = \frac{k\phi^p}{\pi^{1/3}(\frac{3}{4})^{2/3}\theta}$.

Neglecting this constant, we can write the allometric scaling relationship more simply as $W \sim G^{\frac{3p}{2}}$. Thus, the allometric scaling exponent $a = \frac{3p}{2}$ should be multiplied by 2/3 to find an estimate of the Rent exponent, p.

Methodological Limitations There are several limitations to the current work. The human networks provide a necessarily coarse-grained sketch of anatomical wiring on the most global scale. Complete neuronal wiring diagrams for the human brain are not likely to become available in the near future. The small size of both the human and *C. elegans* networks particularly affects the estimation of the fractal topological dimension, D_T. In future studies, it will be useful to apply finer grained parcellation templates to human neuroimaging data to improve precision of fractal dimension estimation. However, to date the template used for the DSI dataset (with 1000 ROIs) is the largest published template. The complementary MRI dataset uses the Brodmann area template whose regions are approximately coincident with cytoarchitecturally defined cortical areas [17]. Thus, the two types of networks reported in this study include the most fine-grained view to date of whole brain white matter tract connectivity and the classical cytoarchitecturally constrained view of whole brain gray matter connectivity. We combined both complementary lines of inquiry to assess consistency, replicability, and robustness of our findings across different data modalities. We expected that the global network architecture of the two types of human networks would be largely consistent based on previous work indicating that individual connections making up these global networks are reproducibly predicted by both modalities [18–21].

The use of covariation in morphometric variables as an indirect measure of structural connectivity between brain regions began in the context of assessing the structural dysconnectivity in schizophrenia possibly due to dysplasia [22–24]. The hypothesis is that reciprical afferent connections benefit both neurons involved by providing a mutually trophic effect; therefore, brain regions whose neurons connect to one another are likely to have positively correlated volumes [18, 24]. Several recent studies have provided experimental validation of this hypothesis by comparing pairs of regions with highly correlated morphometric properties to known fiber tracts established using diffusion tensor imaging [18–21] and tract tracing studies [25–27]. The human MRI network construction method used in the present study is based upon this hypothesis, and has been used previously in the context of both health and disease [19, 28, 29]. As opposed to network contruction based on tract tracing or diffusion imaging, this method only indirectly measures connectivity between brain regions (N=104) over subjects (N=259), thus producing a group network (data and results as in [29]). It is therefore not possible to look at individual variation in the properties of this MRI network. The error in the estimation of the Rent exponent in the MRI network, $\hat{p}_{MRI}$, may be smaller than the between-network variability we would measure if we had several MRI networks from multiple groups of 259 healthy controls. However, we are not aware of further available

datasets of this size given that the study this dataset originates from [29] remains the largest of its kind to date.

The diffusion spectrum imaging network contains an inherent distance bias [30], meaning that long distance connections have a lower probability of being included in the network than short distance connections. While Hagmann and colleagues did use a distance bias correction in the preprocessing of these networks, the most complete correction method remains a matter of ongoing debate [30]. It is possible that some distance bias remains in the current dataset which may artefactually decrease the topological dimension, D_T, the Rent exponent, p_{DSI}, and the average wiring length, $\bar{r}$. However, it is not the purpose of the current study to evaluate the available methods for distance bias correction and we have instead used this recently published dataset which represents one of the currently accepted methods for distance bias correction.

This previously published DSI dataset [30] includes data for 5 subjects with 1 subject scanned twice. As such, this dataset is not adequate to assess the inter-scan reliability or inter-subject reliability of the anatomical structural properties we are studying in this work. Recently, it has been shown that similar whole-brain networks derived from functional MEG data have reproducible topological properties [31]. However, a similar study in anatomical networks has not yet been published, and it will be important in future work to describe the reproducibility of network architecture in terms of both topology and physical embedding.

In this work, the distance between any two network nodes was defined as the Euclidean distance between the center of mass of the brain regions (in the human) or neuronal cell bodies (in *C. elegans*). While this definition is currently widely used [19, 28, 29, 32], it provides an indirect estimate and likely an under-estimate of the true length of white matter tracts and axons in these neural systems, which may take convoluted paths to connect a given pair of nodes. Future advances in diffusion imaging may provide us with better length estimates for white matter tracts in the the human while advances in the characterization of neuronal tissue by reconstruction of electron microscopy images may provide us with better estimates of individual axonal pathways.

The placement embedding for the VLSI circuit required the use of simulated annealing. The estimated Rent exponent based on placement, $p = 0.901$, was less optimal than a previously reported Rent's exponent based on partitioning [12]. It is important to be aware that Rent's exponents based on placement and based on partitioning may not provide the same estimation; the partitioning method does not require

simultaneous physical embedding of all gates in the entire system. We have chosen to use the placement embedding technique to make the results most comparable to the *C. elegans* and human brain network results. In a similar vein, it is important to note that we used the formalism of graphs and edges rather than hypergraphs and hyperedges. The latter are often used in the analysis of VLSI circuits but the concepts are not simply transferable to the biological networks studied here. Thus we have chosen to use simple edges in all reported analyzes to facilitate comparability across systems.

The relationship between allometric scaling and Rentian scaling could be further supported by studying Rentian scaling in MRI or DSI/DTI datasets from a range of mammalian species rather than the human alone. In particular, it would be interesting to discern whether there is a difference in isometric Rentian scaling between mammalian and non-mammalian species as well as between marine and terrestrial mammals who arguably show distinct volumetric scaling relationships [33, 34]. The construction of a comparable species-dependent MRI network would require structural scans from over 200 animals in that species. While no such data is currently available or likely to become available in the near future, the application of DTI specifically to the macaque monkey is a pressing line of current inquiry.

Table 1. Hierarchical modularity in computational and nervous systems for the VLSI (*Column 1*), *C. elegans* (*Column 2*), and human brain (MRI, *Column 3*; DSI, *Column 4*). *Top Panel* Modularity of the entire observed network and comparable functional random and pure random networks. *Second Panel* Number of hierachical levels found in the multi-layer decomposition of the Louvain community detection algorithm. *Third Panel* Number of modules found in the first-level decomposition and their average modularity (*Fourth Panel*). All functional random networks retained the same degree distribution as the observed network; all pure random networks were pure random networks that did not retain the degree distribution of the observed network. Means and standard deviations are over 100 random network instantiations.

Network	VLSI	*C. elegans*	Human brain (MRI)	Human brain (DSI)
**Single Layer Decomposition**				
Modularity				
Observed	0.51	0.40	0.24	0.59±0.02
Random, functional	0.23±0.01	0.20±0.01	0.19±0.01	0.10±0.01
Random, pure	0.17±0.01	0.20±0.01	0.18±0.01	0.15±0.00
**Multi-Layer Decomposition**				
Number of Levels				
Observed	4	3	2	3
Random, functional	4±0	2.86±0.34	2.35±0.47	2.25±0.43
Random, pure	4.95±0.21	3.01±0.10	2.80±0.40	2.98±0.14
First Level Stats				
Number of Modules				
Observed	6	3	7	9
Random, functional	3.23±0.96	4.22±4.50	7.82±4.38	7.00±0.00
Random, pure	15.02±3.27	4.53±1.41	3.92±2.98	3.62±1.85
Average Modularity				
Observed	0.50	0.30	0.14	0.50
Random, functional	0.50±0.01	0.21±0.01	0.14±0.02	0.15±0.01
Random, pure	0.45±0.03	0.23±0.01	0.06±0.03	0.16±0.01

References

1. Sales-Pardo M, Guimerá R, Moreira AA, Amaral LAN (2007) Extracting the hierarchical organization of complex systems. Proc Natl Acad Sci USA 104: 15224-15229.

2. Blondel VD, Guillaume JL, Lambiotte R, Lefebvre E (2008) Fast unfolding of communities in large networks. J Stat Mech : P10008.

3. Maslov S, Sneppen K (2002) Specificity and stability in topology of protein networks. Science 296: 910–913.

4. Reda S (2009) Using circuit structural analysis techniques for networks in systems biology. In: SLIP '09: Proceedings of the 11th international workshop on System level interconnect prediction. New York, NY, USA: ACM, pp. 37–44. doi:http://doi.acm.org/10.1145/1572471.1572478.

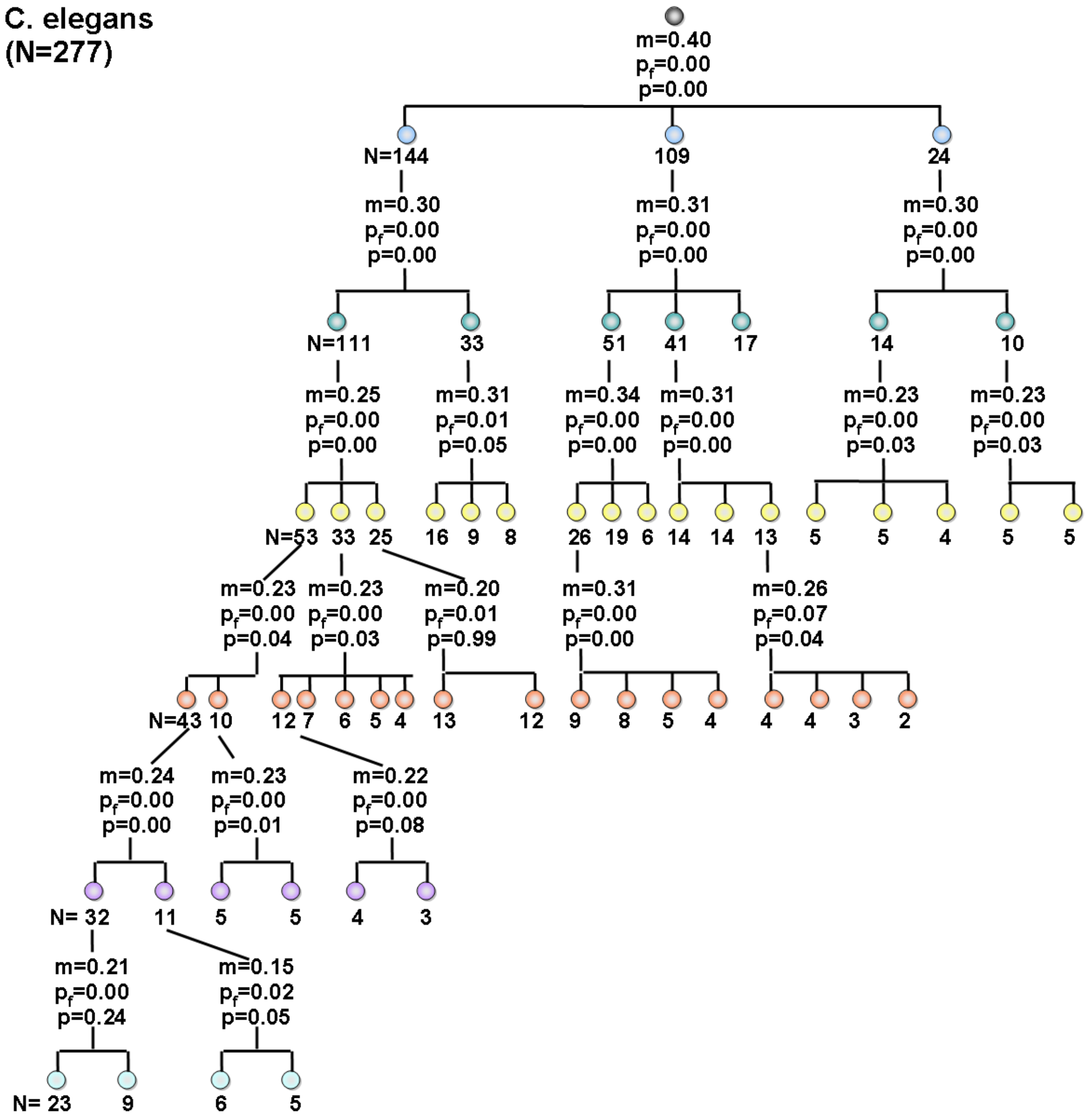

Figure 1. **Full hierarchical modularity decomposition for the *C. elegans* neuronal network** Dendrogram displays the significant modular and sub-modular structure for the neuronal system of the nematode worm. The modularity, m, of each of these matrices was estimated using the Louvain community detection algorithm [2]; p-values for 1-tailed t-tests indicate where the modularity of the observed network was higher than the modularity of a functional random (p_f), and pure random (p) network. The matrices were decomposed into their sub-modules, and each sub-module was tested for modularity, m, greater than functional and pure random networks (p_f, p) of the same size as the module being tested. This process was iteratively performed: sub-modules were tested for non-random modularity, and if sub-sub-modules were identified in this way then each of them was in turn tested for non-random modularity.

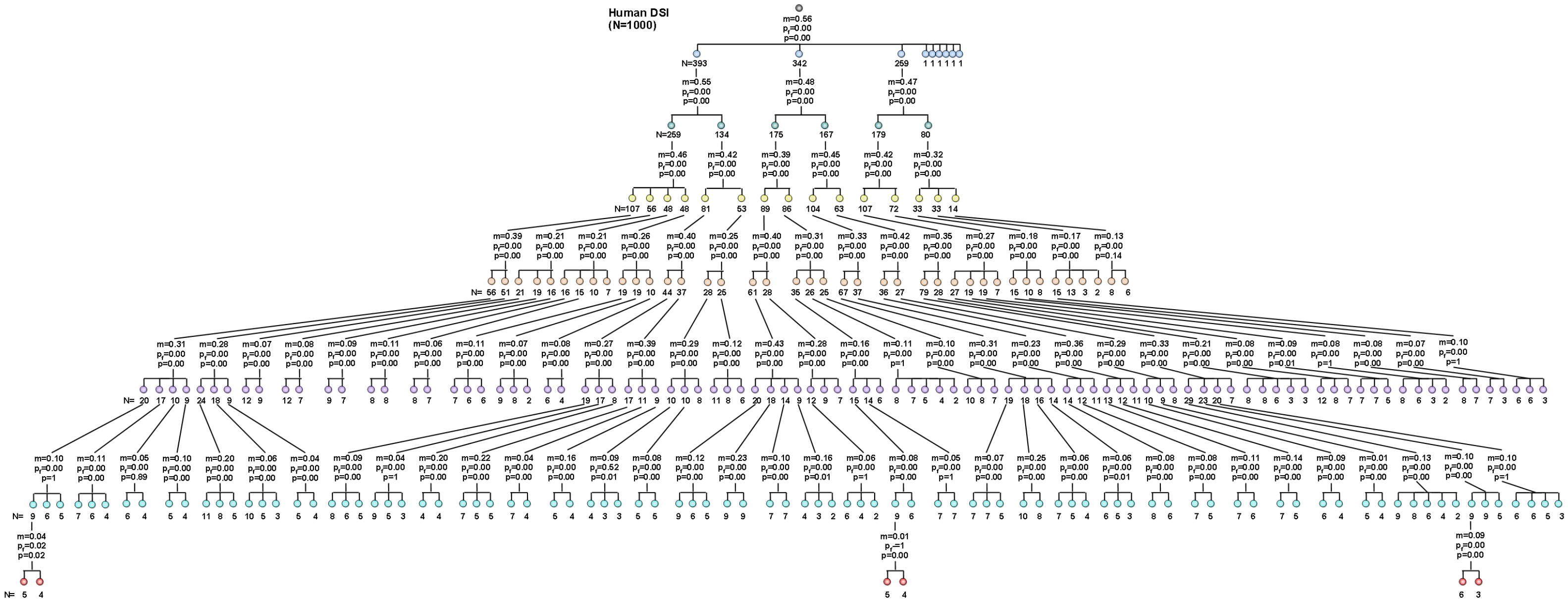

Figure 2. Full hierarchical modularity decomposition for the human anatomical network measured by DSI Dendrogram displays the significant modular and sub-modular structure for the human anatomical network derived from diffusion spectrum imaging (DSI) data. The modularity, m, of each of these matrices was estimated using the Louvain community detection algorithm [2]; p-values for 1-tailed t-tests indicate where the modularity of the observed network was higher than the modularity of a functional random (p_f), and pure random (p) network. The matrices were decomposed into their sub-modules, and each sub-module was tested for modularity, m, greater than functional and pure random networks (p_f, p) of the same size as the module being tested. This process was iteratively performed: sub-modules were tested for non-random modularity, and if sub-sub-modules were identified in this way then each of them was in turn tested for non-random modularity. The decomposition shown in this figure is taken from the first scan of the first subject in the DSI dataset.

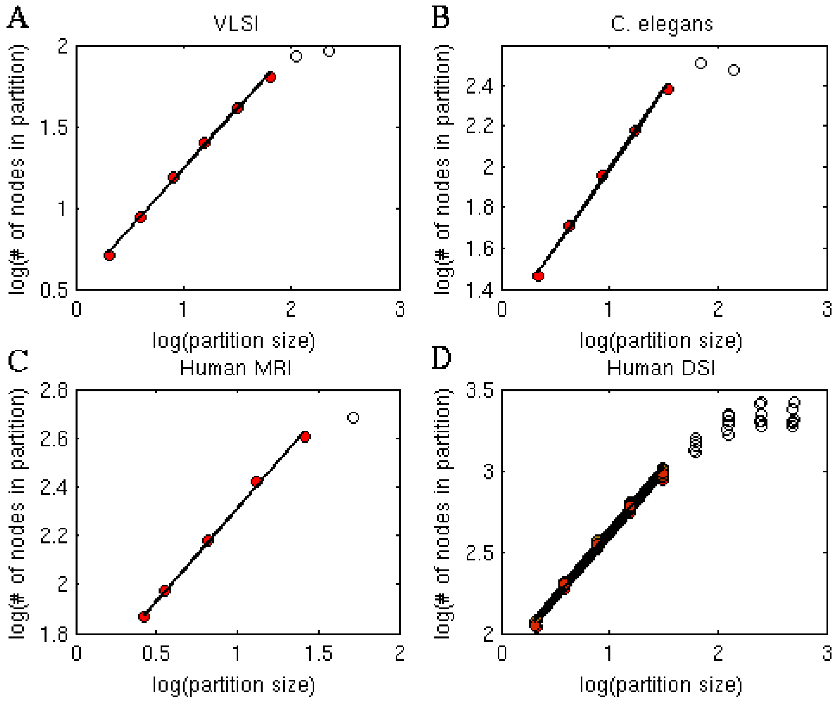

Figure 3. **Topological Rentian scaling in the observed VLSI, *C. elegans* and human brain networks** The network topology of each system was iteratively partitioned in topological space. The number of nodes found in each partition was plotted in log-log space as a function of the partition size. All networks contained a linear scaling regime (so-called Region I, filled circles) and a regime at larger partition sizes where linear scaling broke down due to boundary effects (so-called Region II, empty circles). The slope, p_T, of the line through points within Region I was estimated using a weighted linear regression; see Table 3. Note: Data and linear fits for all six DSI scans are shown in *D*.

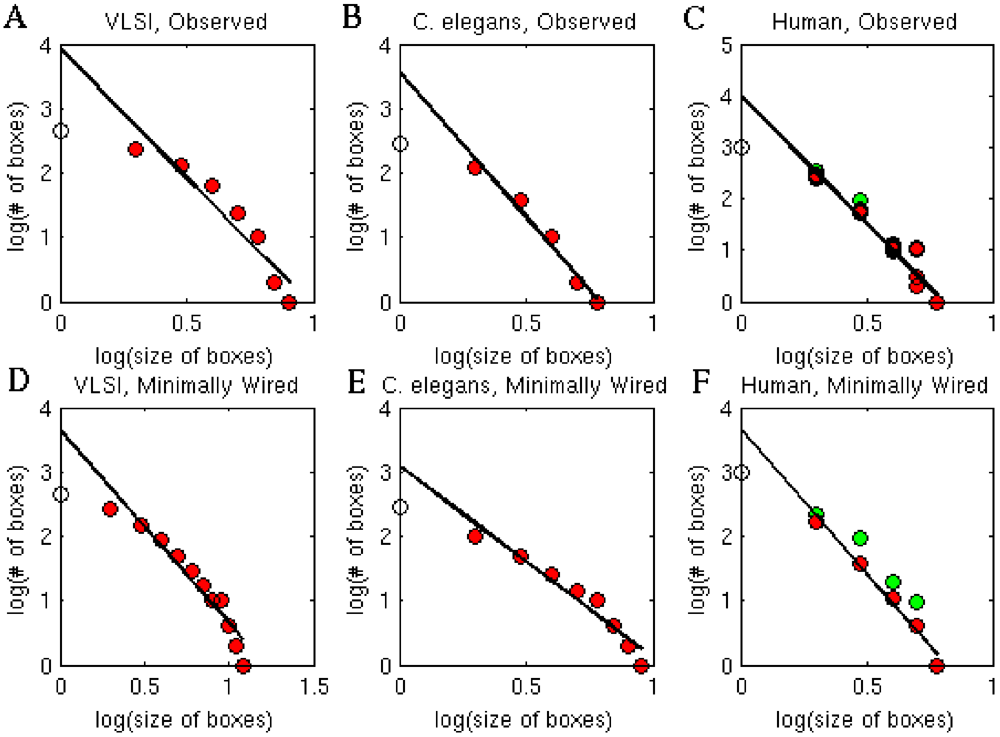

Figure 4. **Box-Counting Fractal Dimension of the Observed and Minimally Wired VLSI, *C. elegans* and Human Brain Networks** The fractal dimension of the observed (*Top Row*) and minimally wired (*Bottom Row*) VLSI circuit (*A,D*), *C. elegans* (*B,E*) and Human (*C,F*) networks is calculated as the slope of the weighted linear regression (using all points except the first, e.g., all filled points) on the number of boxes versus the size of the boxes determined based on the box-counting algorithm of Concas et al. [8]. Human datasets derived from magnetic resonance imaging (MRI) given in green [29] and from a single subject's diffusion spectrum imaging (DSI) given in red [30].

A

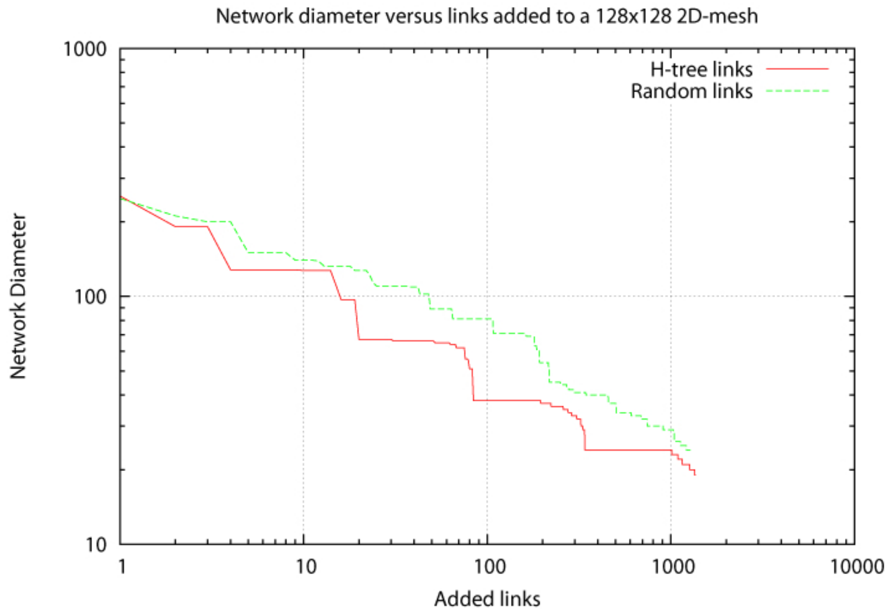

B

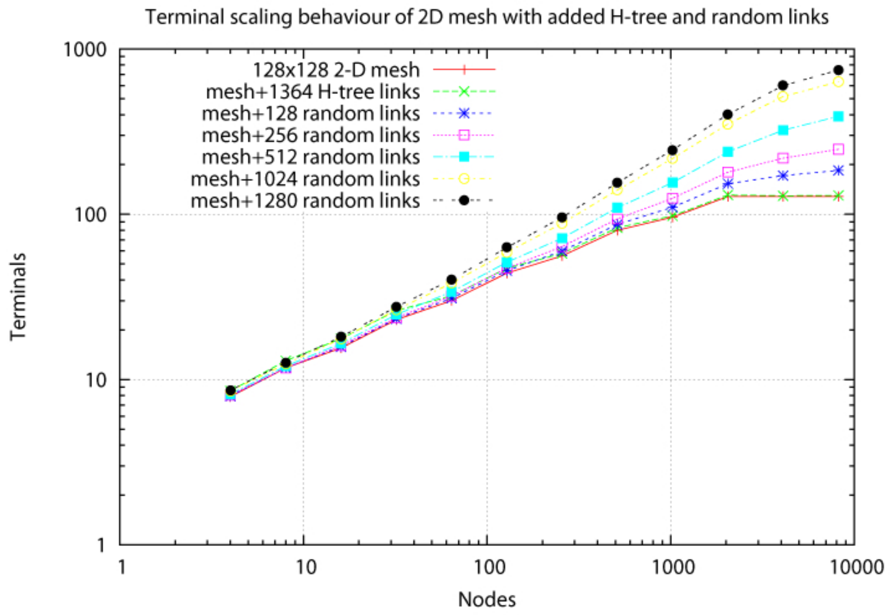

Figure 5. **Dependence of Topology on Addition of Random and H-tree Links** *A* Plot of the network diameter as a function of the number of random (green) or H-tree (red) links added to a 128x128 2D-mesh. *B* Plot of topological Rentian scaling, i.e., the number of terminals as a function of the number of nodes for varying number of random and H-tree links added to a 128x128 2D mesh.

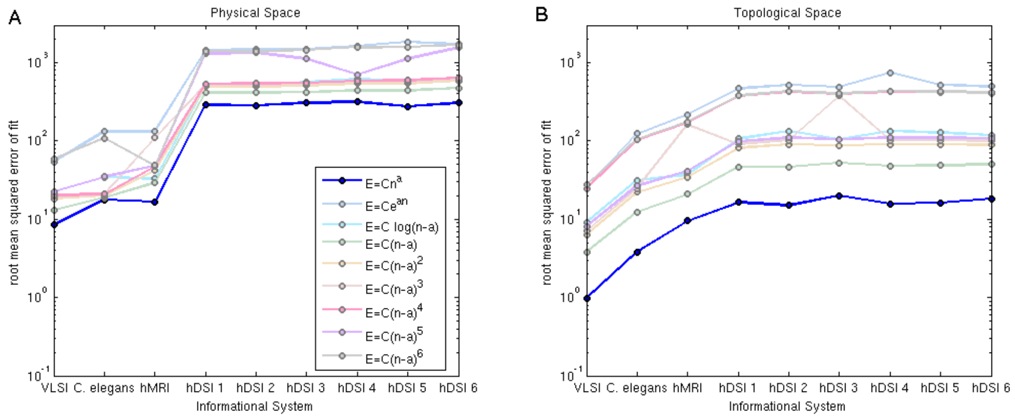

Figure 6. Goodness of Fit in Modeling the Rentian Relationship in physical (A) and topological space (B) between the number of nodes, n, in a partition and the number of edges crossing the boundary of a partition, E. Nine different models were tested (see legend), including the power-law, exponential, logarithmic, linear, and higher order polynomials. All fits contained 2 free parameters: C and a. Here the goodness of fit, as measured by the root mean squared error, is plotted (on the y-axis) for each informational system (x-axis). Note: hMRI = human MRI network, hDSI 1 = human DSI network scan 1, hDSI 2 = human DSI network scan 2, etc.

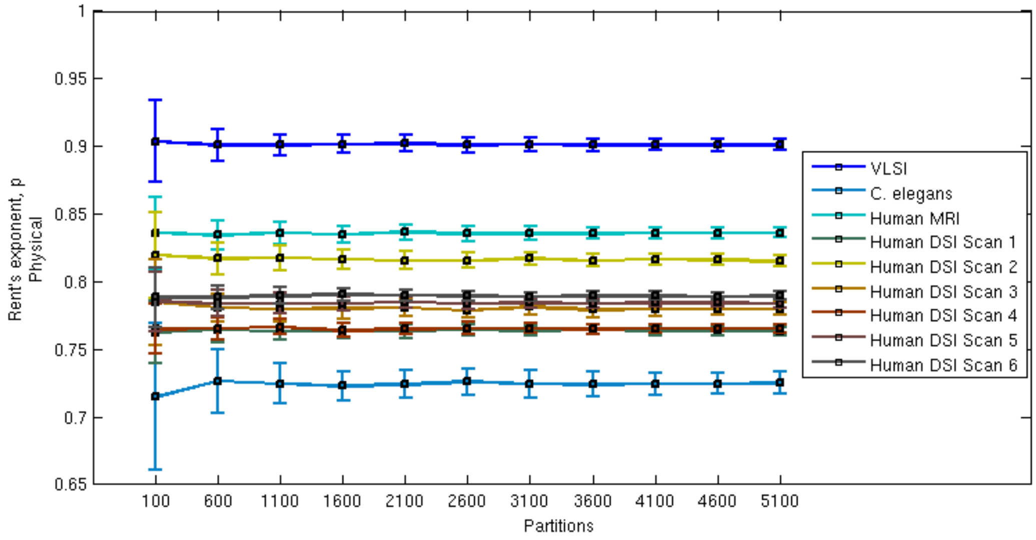

Figure 7. Error Associated With the Number of Partitions and Random Sampling Plot of the estimated physical rent's exponent for all systems under study as a function of the number of partitions (x-axis); error bars indicate standard deviation over 100 resamplings.

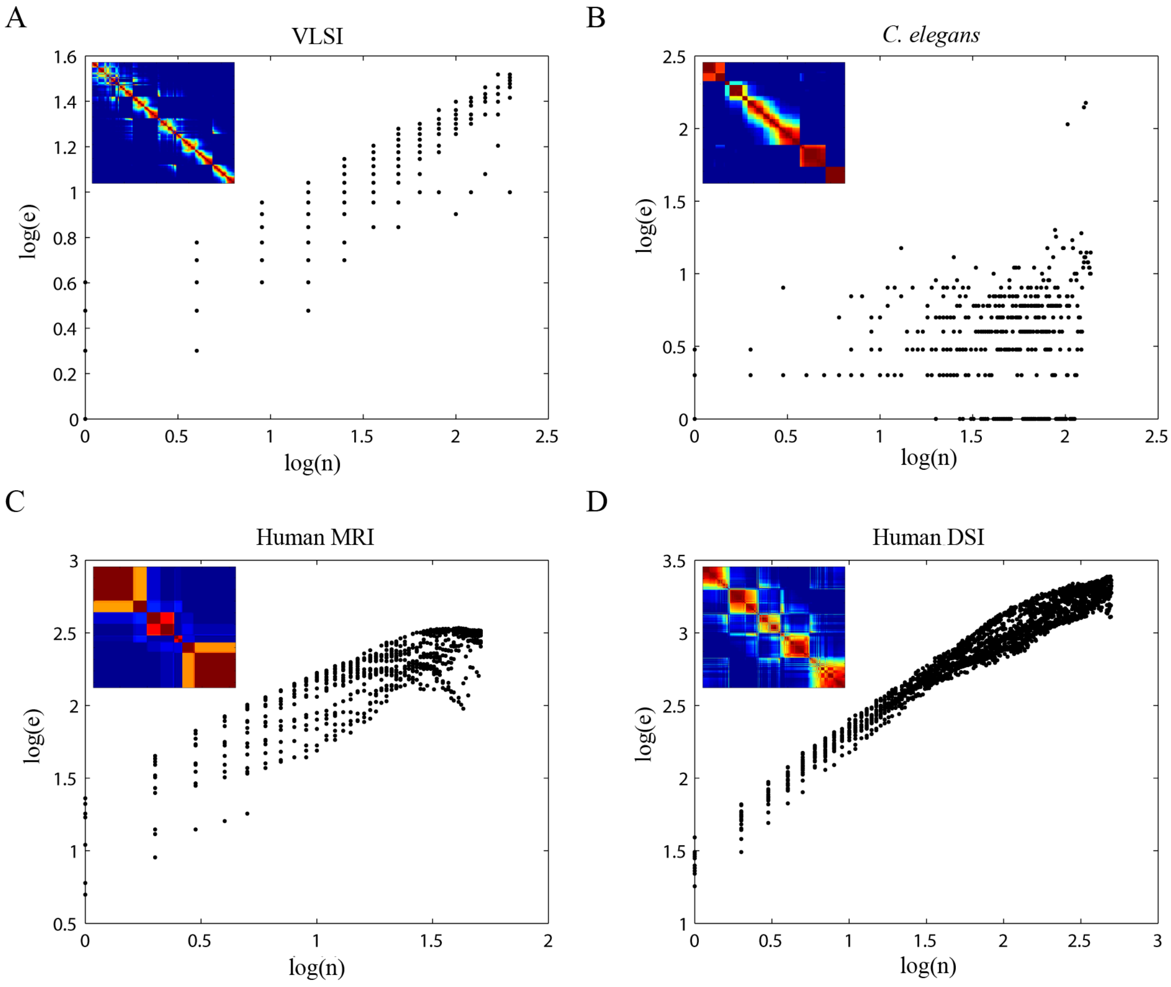

Figure 8. Rentian Characteristics of Minimally Wired Versions of a VLSI (*A*), *C. elegans* (*B*) and the human (*C*-*D*) as estimated from a group network using magnetic resonance imaging (*C*), and a single subject's network using diffusion spectrum imaging (*D*). A least squares regression was used to construct the best fit line where the exponent of the VLSI was found to be $p = 0.509 \pm 0.005$ (with $p = 0.5$ expected for a 2D graph homogeneously embedded in 2D), and for the human was found to be $p = 0.93 \pm 0.01$ (MRI) and $p = 0.68 \pm 0.004$ (DSI) (with $p = 0.67$ expected for a 3D graph homogeneously embedded in 3D). The characteristics of these scaling relationships are heavily influenced by the degree of heterogeneity of node locations in each system. The lowest Rentian exponents are found in both the VLSI and DSI networks where nodes are equally placed in a 2 or 3 dimensional lattice. The MRI and *C. elegans* networks both have heterogeneous node locations with the highest heterogeneity found in the *C. elegans* system. Because of this heterogeneity, minimally wiring the *C. elegans* system completely eradicates Rentian scaling. The inset panel shows the hierarchical modularity of each system, which has been represented by a co-classification matrix where red/brown colors highlight modules or clusters of nodes with high local interconnectivity and relatively sparse connectivity to nodes in other modules [1].

Table 2. **Hierarchical modularity in DSI data** for Subject 1, scan 1 (*Column 1*), Subject 1, scan 2 (*Column 2*), and Subjects 2 through 5 (*Columns 3-6. Top Panel* Modularity of the entire observed network and comparable functional random and pure random networks. *Second Panel* Number of hierachical levels found in the multi-layer decomposition of the Louvain community detection algorithm. *Third Panel* Number of modules found in the first-level decomposition and their average modularity (*Fourth Panel*). All functional random networks retained the same degree distribution as the observed network; all pure random networks were pure random networks that did not retain the degree distribution of the observed network. Means and standard deviations are over 100 random network instantiations.

DSI Data	Subject 1	Subject 1	Subject 2	Subject 3	Subject 4	Subject 5
**Single Layer Decomposition**						
Modularity						
Observed	0.56	0.56	0.62	0.58	0.60	0.60
Random, functional	0.10±0.00	0.10±0.00	0.11±0.00	0.10±0.00	0.10±0.00	0.10±0.00
Random, pure	0.15±0.00	0.15±0.00	0.15±0.00	0.15±0.00	0.15±0.00	0.15±0.00
**Multi-Layer Decomposition**						
Number of Levels						
Observed	3	3	3	3	3	3
Random, functional	2.25±0.43	2.11±0.31	2.17±0.37	2.09±0.28	2.08±0.27	2.16±0.36
Random, pure	2.98±0.14	2.99±0.10	2.98±0.14	2.94±0.23	2.98±0.14	2.99±0.10
First Level Stats						
Number of Modules						
Observed	9	16	23	6	7	11
Random, functional	7.00±0.00	14.0±0.0	21.0±0.0	5.00±0.00	5.00±0.00	9.00±0.00
Random, pure	3.62±1.85	3.27±2.131	3.50±2.12	3.98±3.65	3.59±2.17	3.40±1.27
Average Modularity						
Observed	0.50	0.46	0.50	0.52	0.46	0.46
Random, functional	0.15±0.01	0.14±0.01	0.14±0.00	0.14±0.00	0.14±0.00	0.14±0.01
Random, pure	0.16±0.01	0.15±0.01	0.15±0.01	0.15±0.01	0.15±0.00	0.15±0.00

Network	Partitioning		Box Counting
	p_T	$\widehat{D}_T\,(p_T)$	D_T
VLSI	0.73±0.04	3.81±1.04	4.02±0.66
C. elegans	0.77±0.06	4.42±1.06	4.52±0.49
Human MRI	0.75±0.07	4.12±1.07	5.07±1.58
Human DSI (1)	0.78±0.07	4.54±1.07	4.68±0.77
Human DSI (2)	0.80±0.06	5.06±1.06	4.59±0.76
Human DSI (3)	0.77±0.09	4.42±1.08	4.56±0.81
Human DSI (4)	0.79±0.06	4.97±1.06	5.24±0.39
Human DSI (5)	0.79±0.07	4.84±1.07	4.60±0.82
Human DSI (6)	0.78±0.08	4.73±1.09	4.63±0.76

Table 3. **Topological Rentian Scaling and Fractal Topology**. The topological Rent's exponent, p_T, was estimated using a topological partitioning algorithm implemented in hMetis, version 1.5. The slope of the log of the number of nodes in a partition versus the log of the partition size was estimated using a weighted linear regression; errors indicate standard error of coefficient estimates. Since $p_T \geq p_{min} = 1 - \frac{1}{D_T}$, we estimated the fractal dimension, $\widehat{D}_T\,(p_T)$, implied by this topological rent's exponent (see Column 3). In a complimentary analysis, we directly estimed D_T using a box counting algorithm similar to Concas et. al [8] (see Column 4).

Subject	p	d	$\bar{r}$	κ
1	0.770±0.004	31.0±24.6	4.28	1.87
2	0.807±0.005	37.5±33.3	5.17	2.32
3	0.783±0.006	30.7±25.9	4.24	1.92
4	0.768±0.004	33.4±27.9	4.61	1.72
5	0.780±0.004	32.3±25.9	4.47	2.00
6	0.786±0.004	32.8±25.2	4.53	2.01

Table 4. Individual measures of physical embedding of human DSI networks. p, observed Rent exponent; d length (mean and standard deviation) of connections in mm; $\bar{r}$, mean connection distance between nodes; κ, measure of cost-efficient embedding for the five subjects available in the DSI dataset provided at the Brain Connectivity Toolbox http://sites.google.com/a/brain-connectivity-toolbox.net/bct/. Note: Scans 1 and 2 are from the same subject.

5. Ozaktas HM (1992) Paradigms of connectivity for computer circuits and networks. Opt Eng 31: 1563-1567.

6. Partzsch J, Schüffny R (2009) On the routing complexity of neural network models - Rents Rule revisited. ESANN'2009 proceedings .

7. Karypis G, Kumar V (1999) Multilevel k-way hypergraph partitioning. In: Proc. Design Automation Conference. pp. 343-348.

8. Concas G, Locci MF, Marchesi M, Pinna S, Turnu I (2006) Fractal dimension in software networks. Europhys Lett 76: 1221-1227.

9. Song C, Havlin S, Makse HA (2005) Self-similarity of complex networks. Nature 433: 392-395.

10. Rozenfeld HD, Song C, Makse HA (2010) Small-world to fractal transition in complex networks: A renormalization group approach. Phys Rev Lett 104: 025701.

11. Cancho RFi, Janssen C, Solé RV (2001) Topology of technology graphs: Small world patterns in electronic circuits. Phys Rev E 64: 046119.

12. Stroobandt D (1999) On an efficient method for estimating the interconnection complexity of designs and on the existence of Region III in Rent's rule. Proceedings of the Ninth Great Lakes Symposium on VLSI : 330-331.

13. Burkis J (1991) Clock tree synthesis for high performance ASICs. Proceedings Fourth Annual IEEE International : P9 – 8/1–3.

14. Stroobandt D (2000) Recent advances in system-level interconnect prediction. IEEE Circuits and Systems Society Newsletter 11: 4–20.

15. Prothero JW (1997) Cortical scaling in mammals: A repeating units model. J Brain Res 38: 195-207.

16. Abeles M (1991) Corticonics. Cambridge University Press.

17. Brodmann K (1909) Vergleichende lokalisationslehre der grosshirnrinde .

18. Lerch JP, Worsley K, Shaw GP, Greenstein DK, Lenroot RK, et al. (2006) Mapping anatomical correlations across cerebral cortex (MACACC) using cortical thickness from MRI. NeuroImage 31: 993-1003.

19. He Y, Chen ZJ, Evans AC (2007) Small-world anatomical networks in the human brain revealed by cortical thickness from MRI. Cereb Cortex 17: 2407-2419.

20. Bernhardt BC, Worsley KJ, Besson P, Concha L, Lerch JP, et al. (2008) Mapping limbic network organization in temporal lobe epilepsy using morphometric correlations: insights on the relation between mesiotemporal connectivity and cortical atrophy. Neuroimage 42: 515-24.

21. Bernhardt BC, Rozen DA, Worsley KJ, Evans AC, Bernasconi N, et al. (2009) Thalamo-cortical network pathology in idiopathic generalized epilepsy: insights from MRI-based morphometric correlation analysis. Neuroimage 46: 373-81.

22. Woodruff PW, Wright IC, Shuriquie N, Russouw H, Rushe T, et al. (1997) Structural brain abnormalities in male schizophrenics reflect fronto-temporal dissociation. Psychol Med 27: 1257–66.

23. Bullmore ET, Frangou S, Murray RM (1997) The dysplastic net hypothesis: an integration of developmental and dysconnectivity theories of schizophrenia. Schizophr Res 28: 143-156.

24. Bullmore ET, Woodruff PW, Wright IC, Rabe-Hesketh S, Howard RJ, et al. (1998) Does dysplasia cause anatomical dysconnectivity in schizophrenia? Schizophr Res 30: 127-35.

25. Insausti R, Amaral DG, Cowan WM (1987) The entorhinal cortex of the monkey: II. Cortical afferents. J Comp Neurol 264: 356-95.

26. Jones EG, Powell TP (1970) Connexions of the somatic sensory cortex of the rhesus monkey. 3. Thalamic connexions. Brain 93: 37-56.

27. Houser CR (1992) Morphological changes in the dentate gyrus in human temporal lobe epilepsy. Epilepsy Res Suppl 7: 223-34.

28. He Y, Chen Z, Evans A (2008) Structural insights into aberrant topological patterns of large-scale cortical networks in Alzheimer's disease. J Neurosci 28: 4756-66.

29. Bassett DS, Bullmore ET, Verchinski BA, Mattay VS, Weinberger DR, et al. (2008) Hierarchical organization of human cortical networks in health and schizophrenia. J Neurosci 28: 9239-9248.

30. Hagmann P, Cammoun L, Gigandet X, Meuli R, Honey CJ, et al. (2008) Mapping the structural core of human cerebral cortex. PLoS Biol 6: e159.

31. Deuker L, Bullmore ET, Smith M, Christensen S, Nathan PJ, et al. (2009) Reproducibility of graph metrics of human brain functional networks. Neuroimage 47: 1460-8.

32. Kaiser M, Hilgetag CC (2006) Non-optimal component placement, but short processing paths, due to long-distance projections in neural systems. PLoS Comput Biol 2: e95.

33. Hofman MA (1985) Size and shape of the cerebral cortex in mammals: I. The cortical surface. Brain Behav Evol 27: 28-40.

34. Hofman MA (1988) Size and shape of the cerebral cortex in mammals: II. The cortical volume. Brain Behav Evol 32: 17-26.
